# Non-linear associations between blood pressure and clinical outcomes in atrial fibrillation: a systematic review and dose-response meta-analysis

**DOI:** 10.1093/europace/euag223

**Published:** 2026-08-28

**Authors:** Elnaz Shahmohamadi, Alessandra Pina, Adrian D Elliott, Jonathan P Ariyaratnam, Melissa E Middeldorp, Prashanthan Sanders

**Affiliations:** Centre for Heart Rhythm Disorders, Adelaide University and Royal Adelaide Hospital, 1 Port Road, Adelaide SA, Adelaide 5000, Australia; Centre for Heart Rhythm Disorders, Adelaide University and Royal Adelaide Hospital, 1 Port Road, Adelaide SA, Adelaide 5000, Australia; Centre for Heart Rhythm Disorders, Adelaide University and Royal Adelaide Hospital, 1 Port Road, Adelaide SA, Adelaide 5000, Australia; Centre for Heart Rhythm Disorders, Adelaide University and Royal Adelaide Hospital, 1 Port Road, Adelaide SA, Adelaide 5000, Australia; Centre for Heart Rhythm Disorders, Adelaide University and Royal Adelaide Hospital, 1 Port Road, Adelaide SA, Adelaide 5000, Australia; Centre for Heart Rhythm Disorders, Adelaide University and Royal Adelaide Hospital, 1 Port Road, Adelaide SA, Adelaide 5000, Australia

**Keywords:** Atrial fibrillation, Dose-response meta-analysis, Hypertension, Risk factor, Mortality, Stroke prevention

## Abstract

**Aims:**

Hypertension carries the highest attributable risk for atrial fibrillation (AF) and strongly influences clinical outcomes such as stroke and death; however, the optimal blood pressure targets in individuals with AF remain poorly defined. We aimed to characterize the dose-response relationship between systolic blood pressure (SBP) and diastolic blood pressure (DBP) and ischaemic stroke, all-cause mortality, and intracranial haemorrhage.

**Methods and results:**

We systematically searched MEDLINE, Embase, and Scopus for studies reporting outcomes in AF stratified by SBP and DBP. A two-stage dose-response meta-analysis used restricted cubic splines to model non-linearity, with within-study correlation handled by the Greenland–Longnecker method. Twenty-three studies (17 cohorts; *n* = 476 750) were included. SBP demonstrated a U-shaped association with all-cause mortality, with the lowest risk at 134 mmHg (relative risks (RR) 0.92, 0.86–0.98 vs. 120 mmHg). DBP exhibited a J-shaped association, with the lowest risk at 83 mmHg (RR 0.99, 0.97–1.02). In contrast to mortality, the risks of ischaemic stroke, intracranial haemorrhage, and stroke or systemic embolism rose progressively with SBP, with a steepening gradient. At 140 vs. 120 mmHg, the RR was 1.51 for intracranial haemorrhage against 1.08 for ischaemic stroke, reaching 1.99 vs. 1.14 at 150 mmHg.

**Conclusion:**

SBP exhibits a U-shaped relationship with all-cause mortality in patients with AF, balancing against a monotonic increase in ischaemic stroke and intracranial haemorrhage at higher pressures. A pragmatic target SBP of 120–130 mmHg with DBP kept above ∼75–80 mmHg, appears to optimize the balance between stroke prevention, bleeding risk, and survival.

## Graphical Abstract

**Graphical Abstract:**
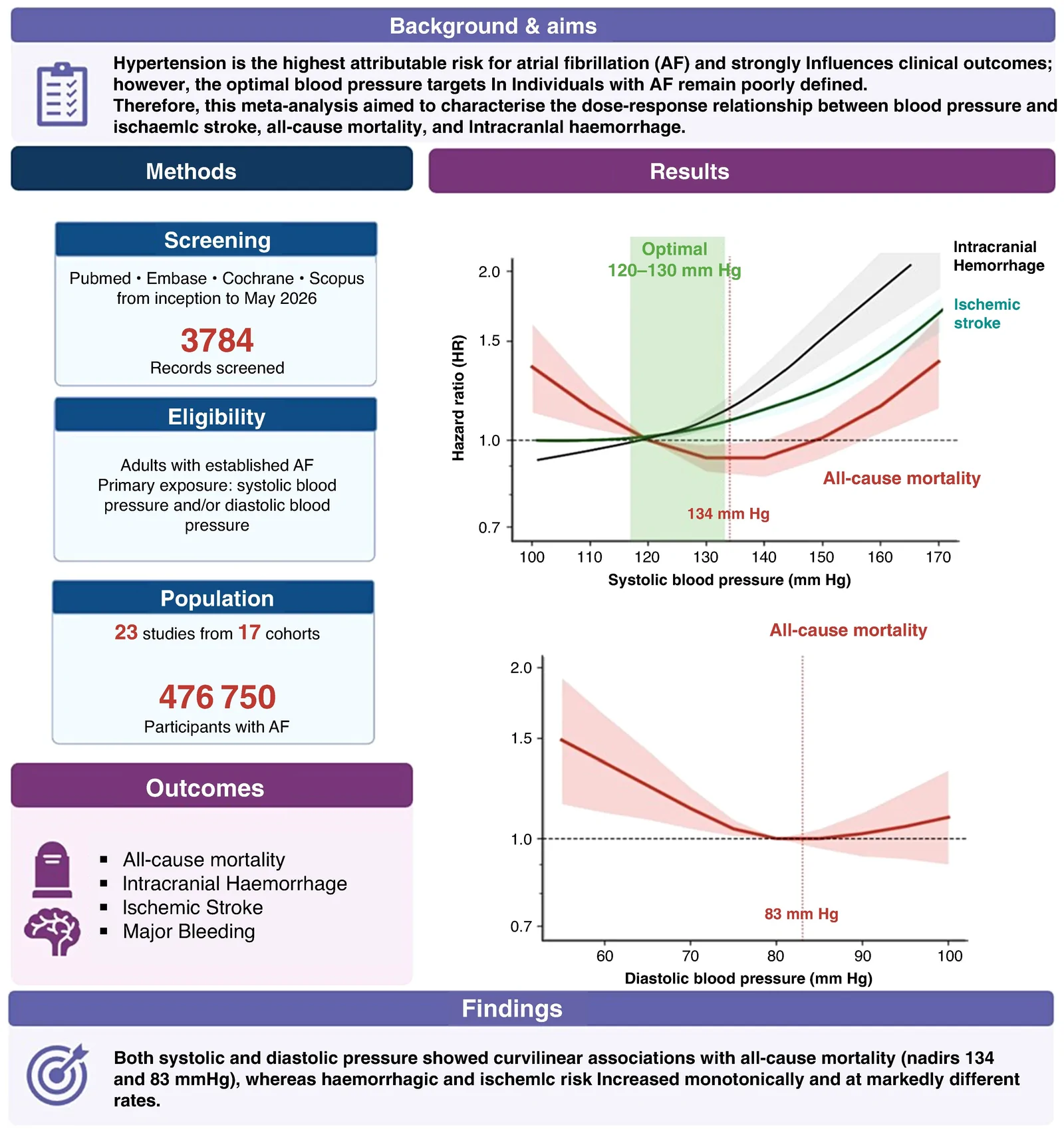

What’s new

- What is the optimal blood pressure range in individuals with atrial fibrillation to reduce the risk of stroke, intracranial haemorrhage, and death?
- Mortality demonstrated a U-shaped for systolic blood pressure (lowest at 134 mmHg), and J-shaped with diastolic pressure (lowest at 83 mmHg). Stroke and intracranial haemorrhage rose steadily with rising systolic BP.
- A systolic BP target of 120–130 mmHg with diastolic BP kept above 75–80 mmHg, may best balance ischaemic, haemorrhagic, and mortality risk in anticoagulated AF patients; lower targets warrant caution given the low-BP harm signal associated with mortality.

## Introduction

Atrial fibrillation (AF) is the most prevalent sustained cardiac arrhythmia worldwide, affecting an estimated 59 million individuals globally.^1–3^ AF is independently associated with a two- to five-fold increased risk of ischaemic stroke, and increased risk of heart failure, all-cause mortality, and substantial rates of cognitive decline and dementia.^4,5^ Hypertension carries the highest population-attributable fraction among modifiable risk factors for incident AF and drives adverse outcomes through atrial structural remodelling, left ventricular hypertrophy, diastolic dysfunction, and endothelial injury.^6,7^

Despite its high prevalence, current clinical guidelines recommend the optimal blood pressure (BP) target in patients with AF extrapolated from general hypertension trials.^8–10^ Crucially, the AF population was systematically under-represented or entirely excluded from landmark BP intervention trials such as SPRINT and ACCORD, leaving a critical evidence gap.^11^ Compounding this, uncontrolled hypertension substantially increases the risk of haemorrhagic events, creating a therapeutic paradox given the concurrent need for oral anticoagulation (OAC) to prevent ischaemic events. This issue cannot be adequately addressed without a comprehensive evaluation of risk patterns across the full spectrum of BPs ranges.

Although randomized controlled trials (RCTs) provide criterion-standard evidence for causal inference, they are inherently constrained to pre-defined treatment targets and are underpowered to characterize risk patterns at the extremes of the BP distribution. Observational studies may offer advantages over trials in population generalizability and in the capacity to evaluate less frequent outcomes over longer follow-up. However, single-cohort studies have reported inconsistent findings regarding the dose-response relationships between BP and clinical outcomes in patients with AF, and evidence remains sparse for certain outcomes. Moreover, the limited sample size at the lower end of the BP distribution in these cohorts has left a substantial gap concerning the dose-response relationships of lower BP levels with clinical outcomes.

Clarifying the dose-response relationships between BP and a range of clinical outcomes could help refine current prevention and treatment strategies. We conducted a systematic review and dose-response meta-analysis to evaluate the non-linear associations between BP levels and major clinical outcomes in adults with established AF.

## Methods

### Study design and registration

This systematic review and dose-response meta-analysis was conducted and reported in accordance with the Preferred Reporting Items for Systematic Reviews and Meta-Analyses (PRISMA) 2020 statement,^12^ (see Supplementary material online, *Methods S1*) and the meta-analysis of observational studies in epidemiology reporting guideline (see Supplementary material online, *Methods S2*). The review protocol was prospectively registered on the International Prospective Register of Systematic Reviews (PROSPERO; CRD420251128571).

### Eligibility criteria

Study eligibility was specified in advance using the PECOS framework (Population, Exposure, Comparator, Outcome, Study design), ensuring transparent and reproducible study selection (see Supplementary material online, *Methods S3*). We included studies enrolling adults (≥18 years) with established AF of any type (paroxysmal, persistent, long-standing persistent, or permanent), confirmed by electrocardiographic criteria or equivalent clinical documentation. The primary exposure was systolic blood pressure (SBP) and/or diastolic blood pressure (DBP), reported as categorical BP strata or as continuous variables. Where available, time in therapeutic range (TTR) data were also extracted and incorporated. For the net clinical benefit (NCB) analysis, anticoagulant treatment class (NOAC, warfarin, or no anticoagulation) was recorded as a key effect modifier. RCTs, *post-hoc* analyses of RCTs, prospective and retrospective cohort studies, and case-control studies were eligible. For the NCB analysis, Phase III RCTs of NOACs vs. warfarin and their pre-specified or *post-hoc* BP subgroup analyses were specifically sought. Case reports, editorials, letters, and review articles were excluded. No language or date restrictions were applied.

### Outcomes

The primary outcome was all-cause mortality. Secondary outcomes included stroke or systemic thromboembolic events (fatal and non-fatal ischaemic stroke or systemic embolism), cardiovascular mortality, major adverse cardiovascular events (as defined per individual study), kidney function, and cognitive decline or dementia.

For the NCB analysis, the efficacy outcome was the rate of ischaemic stroke or systemic thromboembolism per 100 patient-years, and the primary safety outcome was intracranial haemorrhage (ICH; fatal and non-fatal) per 100 patient-years. Major bleeding (ISTH definition or study equivalent) was the secondary safety outcome. The derived outcome was the NCB of each anticoagulant strategy per BP stratum, calculated as described in the Statistical Analysis section.

### Information sources and search strategy

A comprehensive systematic search was conducted across MEDLINE (via PubMed), Embase (via Ovid), Cochrane, and Scopus. Reference lists of all included studies and relevant systematic reviews were hand-searched to identify additional eligible studies. The search strategy was developed using a combination of MeSH/Emtree controlled vocabulary and free-text terms covering (i) AF, (ii) BP, and hypertension, and (iii) the pre-specified outcomes. The full strategy for each database is provided in Supplementary material online, *Methods S4*.

### Study selection

All retrieved citations were imported into covidence and deduplicated. Two independent reviewers (E.S. and A.P.) screened titles and abstracts against the eligibility criteria, followed by full-text assessment for all potentially eligible articles. Disagreements at both stages were resolved by discussion or third-party arbitration. Reasons for exclusion at full-text stage were documented and are reported in the PRISMA 2020 flow diagram. Where multiple reports were derived from the same underlying cohort, they were included only if they evaluated distinct outcomes in the same population; such reports were never pooled within a single analysis, and each cohort contributed to the overall study population only once.

### Data extraction

Data extraction was performed independently by two reviewers (E.S. and A.P.) using a standardized, piloted digital form. Extracted variables included study characteristics such as first author, publication year, country, design, clinical setting, sample size, follow-up duration, and funding sources. Participant characteristics encompassed mean age, sex distribution, AF type, and comorbidities including heart failure, diabetes, prior stroke or transient ischaemic attack, and chronic kidney disease, alongside baseline anticoagulation use.

Exposure data collection captured SBP and DBP per stratum, BP measurement methodology, and background antihypertensive drug classes. Outcome data extraction focused on event rates and adjusted risk estimates, including hazard ratios (HR), odds ratios (OR), or relative risks (RR), with corresponding 95% confidence intervals (CIs), and endpoint definitions. Finally, NCB-specific data included rates per 100 patient-years of ischaemic stroke or systemic embolism and ICH within each BP stratum and anticoagulant cohort, the specific agent and dosage used, and TTR. For studies reporting outcomes across categorical BP strata, the reference category, stratum mid-points or mean BP, and corresponding risk estimates were extracted to enable dose-response modelling.

### Risk of bias assessment

Risk of bias was assessed independently by two reviewers (ES, AP) using tools appropriate to each study design. RCTs were assessed using the Cochrane Risk of Bias Tool version 2 (RoB 2), evaluating bias arising from the randomization process, deviations from intended interventions, missing outcome data, outcome measurement, and selection of reported results. *Post-hoc* analyses of RCTs were assessed using RoB 2 with additional consideration of multiplicity and *post-hoc* exposure categorization. Observational studies were assessed using the ROBINS-E (risk of bias in non-randomized studies—of exposures) tool. Disagreements were resolved by discussion or third-party adjudication. Risk of bias judgements informed sensitivity analyses and certainty of evidence ratings.

### Statistical analysis

Dose-response associations were modelled by a two-stage random-effects approach using the dosresmeta package. In the first stage, study-specific log relative risks across reported strata were combined with their within-study covariances, approximated by the method of Greenland and Longnecker where stratum event counts and participant numbers were available and assumed independent otherwise. Studies contributed their most fully adjusted estimate where available. With the exception of the SPORTIF III/V analysis, which contributed crude incidence-rate ratios, contributing adjusted models included age and sex or a risk score incorporating these variables. Full covariate sets are reported in Supplementary material online, *Table S2*. Further detail on covariance, exposure assignment and knot placement is given in Supplementary material online, *Methods S5*.

Curvilinear associations were modelled with restricted cubic splines, with three knots at the participant-weighted 10th, 50^th^, and 90th percentiles of the exposure distribution and both tails constrained to linearity. Random effects were specified across all spline coefficients jointly, with the between-study covariance matrix estimated by restricted maximum likelihood. Study-specific coefficients were estimated in the first stage and pooled in the second, so that fitted values are predictions from the pooled model rather than interpolations between reported strata, and between-study heterogeneity reflects heterogeneity in the shape of the dose-response relationship rather than in stratum-specific estimates. The basis was parameterized so that the first term is linear in BP; departure from linearity was assessed by a Wald test on the remaining coefficient. Curves are presented relative to 120 mmHg systolic and 80 mmHg diastolic, with the variance structure anchored at the referent so that the confidence interval collapses to unity there. Analyses were restricted to 90–200 mmHg systolic and 50–110 mmHg diastolic, and models were fitted only for outcomes with at least three contributing cohorts. A curve was classified as U-shaped where risk was significantly elevated at both extremes, J-shaped where elevated at one extreme only, and monotonic where significantly non-linear without an interior minimum; a nadir is reported only where an interior minimum was present. Pooled relative risks per 10 mmHg systolic and 5 mmHg diastolic increase were estimated by random-effects meta-analysis with Hartung–Knapp–Sidik–Jonkman confidence intervals, studies reporting only categorical data contributing a slope from linear dose-response regression. Between-study variance was estimated by restricted maximum likelihood and reported with I², H and Cochran's Q. This analysis was not performed for all-cause mortality, since a single linear slope averages the two limbs of a U-shaped association; heterogeneity among its study-specific slopes is reported descriptively. Leave-one-out influence analyses were performed for every modelled outcome, with knots held at full-model values. The NCB of NOAC vs. warfarin at each stratum was calculated using the Singer–Lip formula, in which the difference in stroke rates is offset by 1.5 times the difference in intracranial haemorrhage rates, both per 100 patient-years (Supplementary material online, *Methods S5*); estimates are presented descriptively. Where at least ten estimates contributed, small-study effects were assessed by contour-enhanced funnel plots, Egger's weighted regression and Begg's rank correlation. Analyses used R 4.3.0 (dosresmeta, metafor), with a two-sided α of 0.05.

### Publication bias and quality of evidence

For outcomes where at least 10 studies contributed to a conventional meta-analysis, small-study effects and publication bias were evaluated using funnel plot asymmetry, Egger’s weighted regression test, and Begg’s rank correlation test. Contour-enhanced funnel plots were generated to differentiate true publication bias from other potential causes of asymmetry, such as between-study heterogeneity.

## Results

An initial database search yielded 3928 records. After removing 1144 duplicates and automated exclusions, 2784 records were screened by title and abstract, leading to the exclusion of 2705 items. The remaining 79 full-text articles were evaluated for eligibility, from which 56 were excluded (reasons detailed in *Figure 1*). Ultimately, 23 studies met all criteria and were included in the final systematic review and meta-analysis.^13–34^

**Figure 1:**
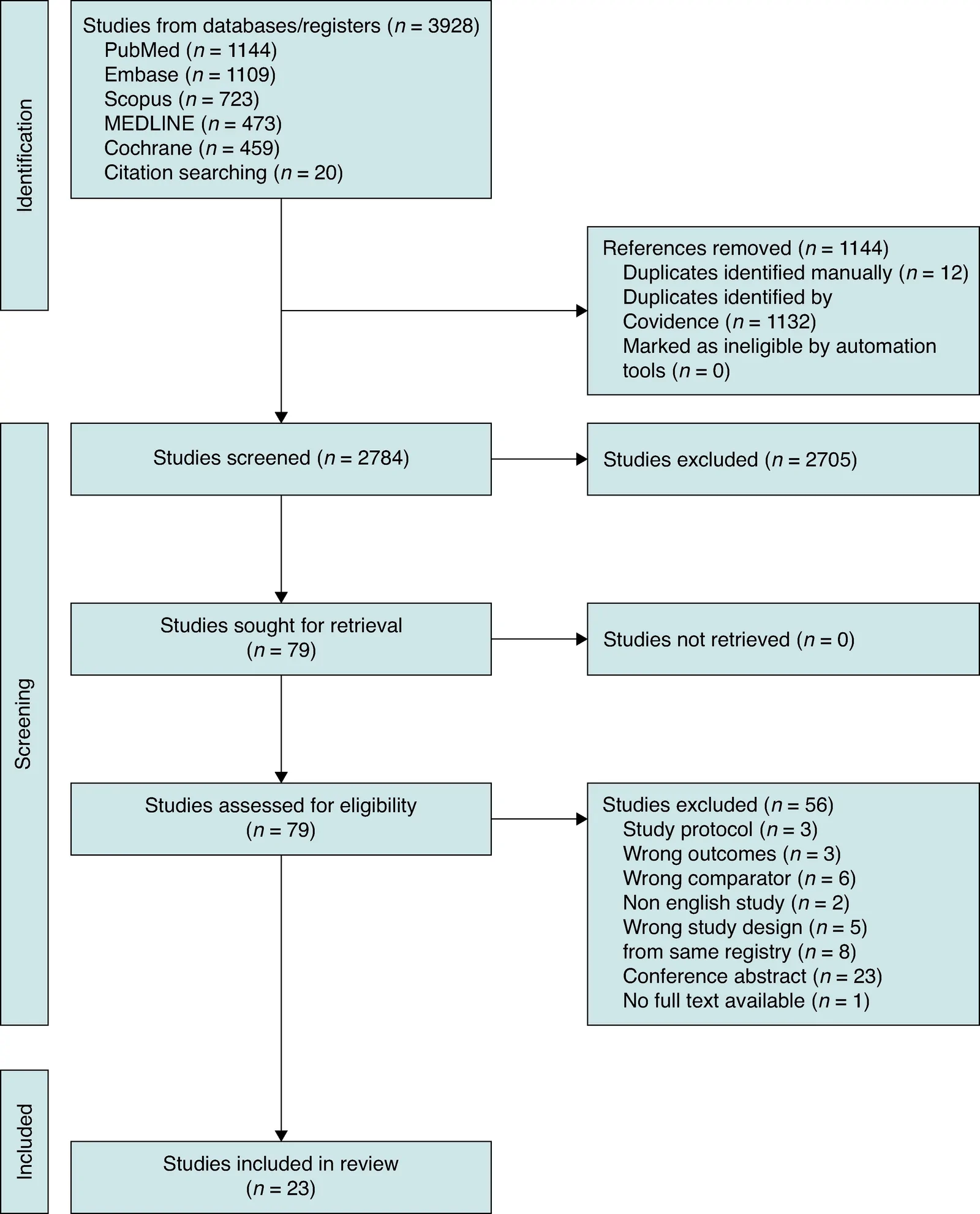
PRISMA 2020 flow diagram.

### Quality assessment results

Overall, 3 of the 23 included studies were assessed at low risk of bias, 17 raised some concerns, and 3 were at high risk. Concerns were most frequently driven by outcome measurement and selection of reported results (Domains 4 and 5, respectively). This primarily reflected non-systematic BP measurement or bias inherent to outcome assessment in observational study designs. Detailed domain-level judgments for each study are provided in Supplementary material online, *Figures S1* and *S2*.

### Study characteristics

A total of 17 cohorts from 23 articles, involving 476 750 participants with AF met the inclusion criteria. The mean follow-up duration ranged from 1 to 6.6 years (*Table 1*). The average age was 70.9 ± 3.2 years. Studies were published between 2014 and 2026. Among the cohorts, 8 reported a prospective study design, and most studies (22/23) reported office BP, one reported home BP. BP measurement methods for each cohort are summarized in Supplementary material online, *Table S1*, and outcome definitions with the corresponding model adjustments in Supplementary material online, *Table S2*. The number of cohorts differed by outcome: All-cause mortality (11 cohorts, 407 885 participants), ischaemic stroke (5 cohorts, 365 161 participants), intracranial haemorrhage (4 cohorts, 169 540 participants), stroke or systemic embolism (4 cohorts, 42 817 participants) and major bleeding (3 cohorts, 43 080 participants). Dementia (1 cohort, 171 228) contributed too little for dose-response modelling and is reported descriptively. The contribution of each cohort to each analysis is shown in Supplementary material online, *Tables S3* and *S4*. All pooled estimates are expressed relative to an SBP of 120 mmHg and DBP of 80 mmHg. Model characteristics and fitted curve shapes are summarized in Supplementary material online, *Table S5*, and pooled estimates per 10 mmHg increment in Supplementary material online, *Table S6*. Three studies reporting the association between SBP-time in target range (SBP-TTR) and clinical outcomes in patients with AF were identified: Kim *et al*.,^33^the COOL-AF registry analysis,^31^ and Kodani *et al*.^22^ and were reported descriptively (see Supplementary material online, *Tables S10*–*S12*).

**Table 1:** Characteristics of included studies

Cohort	Design	Country/region	AF patients (*n*)	Follow-up (years)	Age (mean ± SD)	Female (%)	HTN (%)	OAC Use (%)	OAC type	CV comorbidities (%)
Badheka, 2014^13^	*Post-hoc* analysis of RCT (AFFIRM)	North America	3947	3.3 years	68.6 ± 9.5	24.50%	63.40%	86.70%	Warfarin	CAD: 28.8; HF: 36.0
Bager, 2021^14^	Observational cohort study (SPCCD-SKA)	Sweden	3972	Mean 4.3 years	77.0 ± 8.5	47.80%	100%	100%	Warfarin: 87.8%; Rivaroxaban/Apixaban/Edoxaban: 11.6%; Dabigatran: 1.0%	CAD: 34.0; CVD: 26.4; HF: 29.1; Diabetes: 24.7; PAD: 6.4; COPD: 5.8
Bohm, 2020	*Post-hoc* analysis of RCT (RE-LY Trial)	54 Countries	18 107	Median 2 years	71.5 ± 8.7	36.40%	78.90%	100%	Dabigatran, Warfarin	CAD: 27.8; HF: 32.0
Krittayaphong, 2024^16^	Prospective Registry (COOL-AF)	Thailand	3355	2.7 ± 0.7	67.8 ± 11.2	41.90%	68.60%	75.50%	Warfarin, NOACs	HF: 26.6; CAD: 16.1
Krittayaphong, 2021^31^	Prospective Registry (COOL-AF)	Thailand	3384	3.1 ± 1.5	67.3 ± 11.2	41.40%	67.90%	73.10%	Warfarin, NOACs	HF: 27.2; CAD: 15.6
Ishida, 2026^17^	Retrospective Cohort (F-CREATE)	Japan	4039	Mean 2.80	55.8–59.0	nm	80%	100%	VKA, NOACs	Diabetes: 18.9–35.4
Kodani, 2022^34^	Pooled Registry (J-RISK AF)	Japan	15 019	2	70.0 ± 11.0	30.90%	72%	71.7–74.2%	Warfarin, DOAC	HF: 20–33; Stroke: 14
Kario, 2023^20^	Prospective Registry (ANAFIE)	Japan	4933	1.88	81.4 ± 4.8	43.80%	77%	93%	DOAC (71), VKA (22)	Cardiac: 55; Cerebro: 22
Jeong, 2026^21^	Population Database (NHIS)	South Korea	338 747	Median 6.0	67.4 ± 11.3	41.70%	74.90%	nm	nm	HF: 33.7; Stroke: 19.5
Kim, 2018^18^	Population Database (NHIS)	South Korea	298 374	Mean 5.6 ± 3.5	Median 64 (54–72)	40.7%	79.4%	OAC-naive	OAC-naive	CVD: 32.2%; HF: 20.8%; Diabetes : 17.8%; Previous ischaemic stroke: 13.1%; Previous myocardial infarction: 6.4%
Kim, 2020^19^	Population Database (NHIS)	South Korea	171 228	Mean 9.0	61.7 ± 9.9	45.20%	58.40%	65.20%	Warfarin, NOAC	Stroke: 11.5; HF: 14.2
Kim, 2022^33^	Retrospective Review (Asan Medical)	South Korea	13 532	Median 2.7	65.0 ± 11.9	36.40%	70.20%	59.30%	nm	HF: 19.5; Stroke: 12.1
Lip, 2007^23^	Pooled analysis of RCTs (SPORTIF III and V)	23 Countries & North America	10 763	Median 1.55	71.5 ± 8.5	30.20%	74.10%	100%	Warfarin, Ximelagatran	CAD: 45; HF: 36.5
Park, 2019^26^	*Post-hoc* analysis of RCT (ENGAGE AF-TIMI 48)	54 Countries	19 679	Median 2.8	70.6 ± 9.4	38.60%	100%	100%	Edoxaban, Warfarin	CHF: 57.7; DM: 36.7
Minhas, 2020^27^	Retrospective Cohort (CPRD)	UK	9051	1.6	77.2 ± 10.6	46.60%	62.40%	100%	DOACs	Stroke/IHD: 43.4
Kodani, 2022^22,34^	Prospective Registry (J-RHYTHM)	Japan	7226	2	70 ± 10	29%	61%	87%	Warfarin	CAD: 10; HF: 28
Xu, 2023	Multi-centre Registry (Chinese AF registry)	China	1990	1	Median 71.0	41.20%	55%	16.80%	Warfarin	CAD: 40.5; HF: 35.3
Tremblay-Gravel, 2014^32^	*Post-hoc* analysis of RCT (AFFIRM and AF-CHF)	North America & Europe	5436	Mean 3.4 (41 ± 16 mos)	67.0 ± 10.0	31.80%	76.40%	91.20%	Warfarin	HF: 36.4; CAD: 42.1
Shao, 2022^24^	Prospective Registry (MIMIC-III)	USA	2345	1.0 (Hospital/1-yr)	73.1 ± 12.4	44.10%	71.20%	48.50%	Warfarin, DOAC	HF: 41.2; DM: 28.4
Shantsila, 2024^25^	Prospective Registry (AF-GEN-UK)	UK	1120	Mean 3.2	74.8 ± 11.0	44.60%	78.50%	89.20%	Warfarin, DOAC	Stroke/TIA: 14.2; HF: 22.1
Vemulapalli, 2019^30^	Registry (ORBIT-AF)	USA	10 098	2	73.5 ± 11	42.30%	83%	76.10%	Warfarin (71.2)	CAD: 36.0; HF: 32.1
Vemulapalli, 2016^29^	ROCKET AF	International	14 256	Median 1.94	Median 73	39.8%	90.5%	100%	Warfarin or Rivaroxaban	HF: 62.7%; diabetes : 40.0%;
Hankey 2014^28^	ROCKET AF	International	14 264	Median 1.94	Median 73 years (IQR 65–78	39.7%	90.5%	100%	Warfarin or Rivaroxaban	HF: 62.7%; diabetes : 40.0%; History of stroke or TIA: 51.9%

AF, atrial fibrillation; BP, blood pressure; CAD, coronary artery disease; DOAC, direct oral anticoagulant; HF, heart failure; HTN, hypertension; nm, not mentioned; OAC, oral anticoagulant; RCT, randomized controlled trial; SBP, systolic blood pressure; VKA, vitamin K antagonist.

### All-cause mortality

#### Systolic BP

A U-shaped association was observed between SBP and all-cause mortality (LRT *P* for non-linearity <0.001), with a nadir at SBP of 134 mmHg (RR 0.92; 95% CI, 0.86–0.98) (*Figure 2*). Mortality risk was elevated at both lower SBP (RR 1.35 [1.12–1.61] at 100 mmHg; RR 1.14 [1.05–1.24] at 110 mmHg) and higher SBP (RR 1.15 [1.03–1.30] at 160 mmHg; RR 1.38 [1.14–1.66] at 170 mmHg). Heterogeneity was substantial on both limbs and greater on the low-pressure limb (I² 97%, 95% CI 95–98%) than the high-pressure limb (I² 87%, 95% CI 78–92%). In exploratory subgroup analyses and meta-regression, study design, anticoagulant era, measurement setting and the proportion anticoagulated did not explain this heterogeneity (see Supplementary material online, *Table S18*). Because a single linear slope averages the two limbs of a U-shaped association, a pooled per-10 mmHg estimate was not derived for this outcome (see Supplementary material online, *Table S6*). Leave-one-out sensitivity analysis confirmed that the U-shaped pattern was robust to exclusion of any single study. Removing any single cohort in turn shifted the nadir between 134 and 136 mmHg, against 134 mmHg in the full model (Supplementary material online, *Figure S3*).

**Figure 2:**
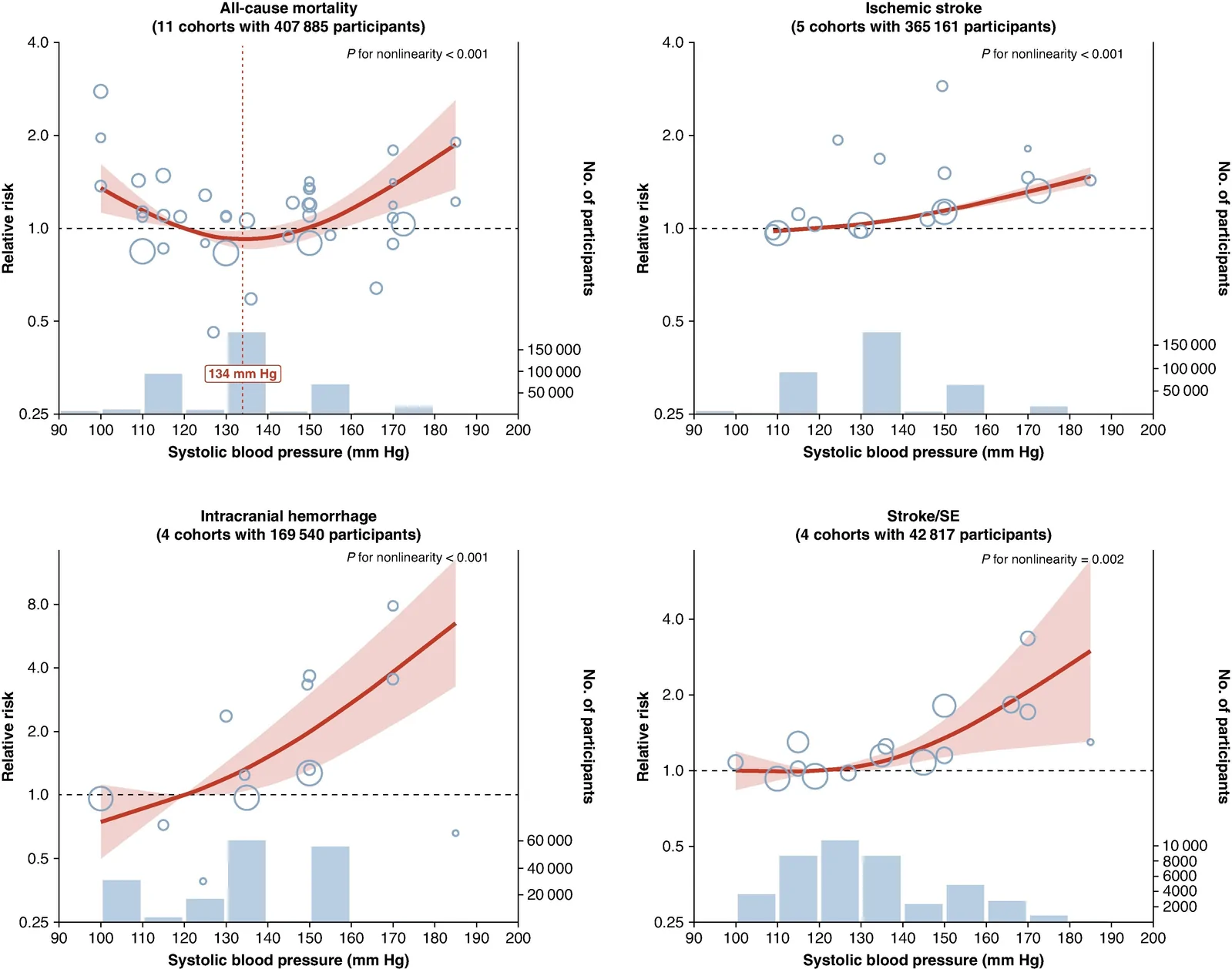
Dose-response meta-analysis of SBP and clinical outcomes in patients with AF.

#### Diastolic BP

A dose-response meta-analysis of six studies including 388 573 patients with AF demonstrated a significant non-linear association between DBP and all-cause mortality (*P* for non-linearity = 0.004). Relative to a diastolic pressure of 80 mmHg, all-cause mortality showed a J-shaped association with a nadir at 83 mmHg (relative risk 0.99; 95% CI, 0.97–1.02). Risk rose progressively at lower diastolic pressures: 1.13 (1.04–1.23) at 70 mmHg, 1.24 (1.08–1.43) at 65 mmHg, 1.36 (1.11–1.65) at 60 mmHg and 1.49 (1.15–1.92) at 55 mmHg and was significantly elevated throughout the range 55 to 76 mmHg (*Figure 3*). Heterogeneity among the study-specific linear slopes was substantial (I² = 92.1%; Q = 63.5, 5 df, *P* < 0.001; τ² = 0.0024). Too few cohorts reported diastolic data for the remaining outcomes to permit dose-response modelling (see Supplementary material online, *Table S8*-*S9*).

**Figure 3:**
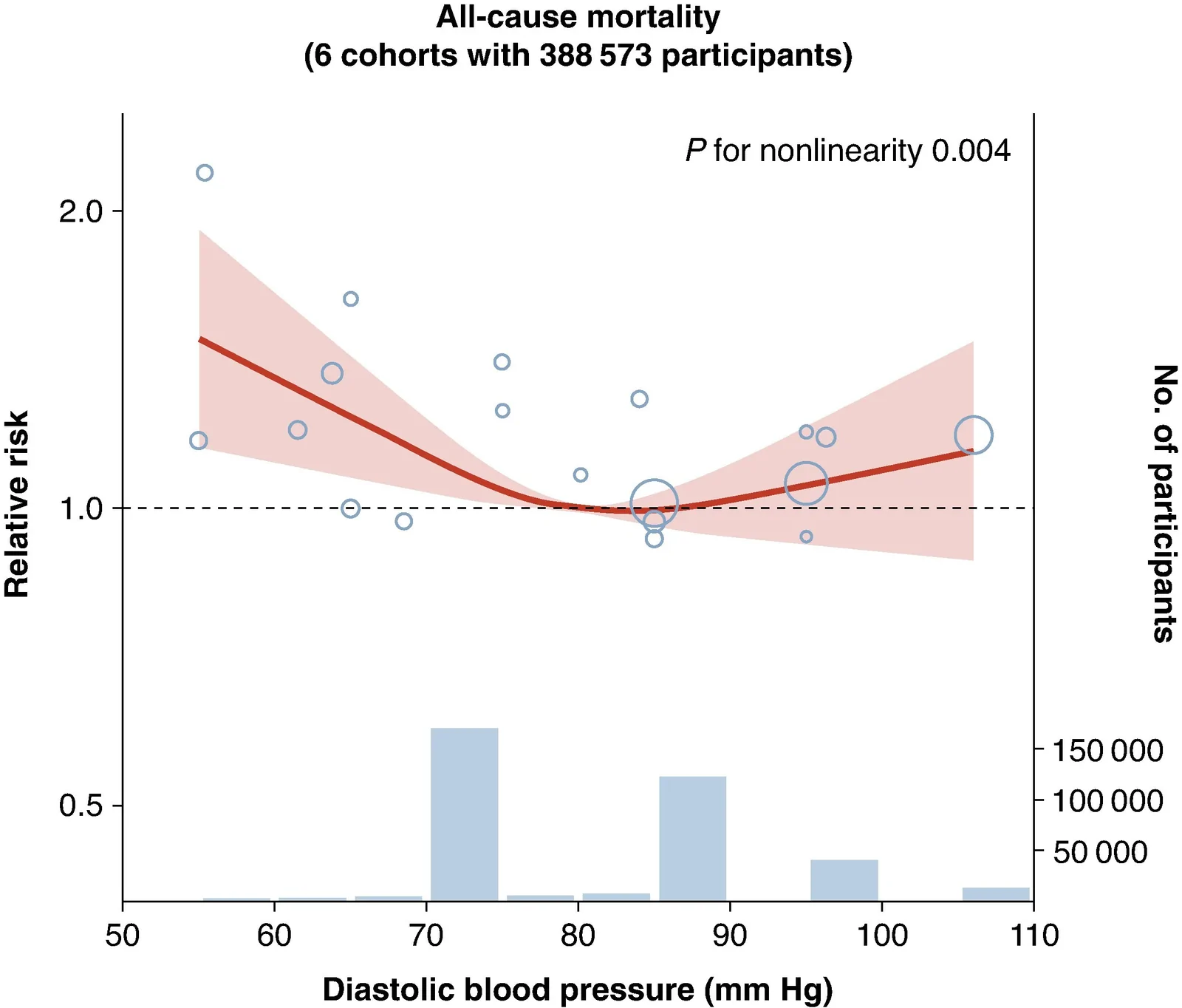
Dose-response meta-analysis of DBP and all-cause mortality in patients with AF.

#### Intracranial haemorrhage

Four cohorts (169 540 participants; 4317 events) contributed. A positive monotonic association was observed between SBP and ICH risk (Wald *P* for non-linearity <0.001) (*Figure 2*). Risk increased progressively above SBP 120 mmHg, reaching RR 1.20 (95% CI, 1.01–1.42) at SBP 130 mmHg, RR 1.99 (1.31–3.00) at SBP 150 mmHg, RR 2.72 (1.65–4.47) at SBP 160 mmHg, and RR 3.82 (2.15–6.79) at SBP 170 mmHg. Risk was not elevated below the referent: 0.86 (0.71–1.04) at 110 mmHg and 0.75 (0.50–1.12) at 100 mmHg. The pooled estimate per 10 mmHg increment was 1.20 (1.01–1.43), with substantial heterogeneity (I² 82%; 95% CI, 43–98), (see Supplementary material online, *Table S6*).

#### Ischaemic stroke

Five cohorts (365 161 participants; 39 966 events) contributed. A positive monotonic association was observed (Wald *P* for non-linearity < 0.001; *Figure 2*). Risk was stable near the reference below SBP 140 mmHg (RR 1.03 (1.02–1.04) at SBP 130 mmHg) and increased significantly at higher SBP: RR 1.14 (1.12–1.16) at 150 mmHg and RR 1.31 (1.26–1.37) at 170 mmHg. Estimates below the referent were close to unity (RR 0.98 [0.97–0.99] at 110 mmHg; RR 0.96 [0.92–1.01] at 100 mmHg). The pooled estimate per 10 mmHg increment was 1.07 (1.00–1.13), with moderate heterogeneity (I² 55%; 95% CI, 0–97) (see Supplementary material online, *Table S6*).

#### Stroke/systemic embolism

Four cohorts (42 817 participants; 1166 events) contributed. The association departed significantly from linearity (Wald *P* = 0.002) and was monotonically increasing (*Figure 2*). Risk did not differ from the referent at 130 mmHg (RR 1.05; 95% CI, 0.99–1.10) and became statistically significant from 140 mmHg (RR 1.15; 1.06–1.26), rising to RR 1.34 (1.13–1.60) at 150 mmHg, RR 1.64 (1.19–2.25) at 160 mmHg, and RR 2.06 (1.24–3.41) at 170 mmHg. Below the referent, estimates were consistent with no association (RR 0.99 [0.91–1.07] at 110 mmHg; RR 1.00 [0.84–1.20] at 100 mmHg). The pooled estimate per 10 mmHg increment was 1.10 (1.05–1.16), with moderate heterogeneity (I² 53%; 95% CI, 0–92; Supplementary material online, *Table S6*).

#### Major bleeding

Three cohorts (43 080 participants; 535 events) were included. The association departed significantly from linearity (Wald *P* = 0.002), with a nadir at 133 mmHg (RR 0.95; 95% CI, 0.82–1.10); however, no level across the modelled range differed significantly from the referent, with estimates spanning unity from RR 1.23 (0.75–2.02) at 100 mmHg, through RR 0.95 (0.83–1.09) at 130 mmHg, to RR 1.22 (0.85–1.74) at 170 mmHg (*Table 2*; Supplementary material online, *Table S5*). With few contributing cohorts, and this outcome should be regarded as inconclusive rather than null. Consistent with this, the pooled estimate per 10 mmHg increment was 1.00 (0.94–1.07), albeit with substantial heterogeneity (I² 73%; 95% CI, 18–98) (see Supplementary material online, *Table S6*).

**Table 2:** Pooled relative risk of each outcome across SBP, referent 120 mmHg

Outcome	100	110	120	130	140	150	160	170
Intracranial haemorrhage	0.75 (0.50–1.12)	0.86 (0.71–1.04)	1.00 (ref)	1.20 (1.01–1.42)	1.51 (1.11–2.05)	1.99 (1.31–3.00)	2.72 (1.65–4.47)	3.82 (2.15–6.79)
Ischaemic stroke	0.97 (0.94–0.99)	0.98 (0.97–0.99)	1.00 (ref)	1.03 (1.02–1.04)	1.08 (1.06–1.09)	1.14 (1.12–1.16)	1.22 (1.19–1.25)	1.31 (1.26–1.37)
Stroke or systemic embolism	1.00 (0.84–1.20)	0.99 (0.91–1.07)	1.00 (ref)	1.05 (0.99–1.10)	1.15 (1.06–1.26)	1.34 (1.13–1.60)	1.64 (1.19–2.25)	2.06 (1.24–3.41)
Major bleeding	1.23 (0.75–2.02)	1.10 (0.88–1.37)	1.00 (ref)	0.95 (0.83–1.09)	0.96 (0.82–1.12)	1.01 (0.89–1.15)	1.10 (0.91–1.34)	1.22 (0.85–1.74)
All-cause mortality	1.35 (1.12–1.61)	1.14 (1.05–1.24)	1.00 (ref)	0.93 (0.88–0.98)	0.93 (0.86–1.00)	1.01 (0.93–1.10)	1.15 (1.03–1.30)	1.38 (1.14–1.66)

Relative risk (95% CI) at fixed SBPs, from two-stage random-effects dose-response models using restricted cubic splines with knots at the participant-weighted 10th, 50th, and 90th percentiles. Columns are SBP in mmHg.

Publication bias could be assessed only for all-cause mortality, the sole outcome with at least ten contributing estimates; neither Egger's test (*P* = 0.35) nor Begg's rank correlation (*P* = 0.54) indicated small-study effects, and trim-and-fill adjustment left the estimate essentially unchanged (see Supplementary material online, *Figure S2*).

#### Analyses informed by single cohorts

Four pre-specified secondary analyses were each informed by a single cohort and could not be pooled; full estimates are provided in Supplementary Results and Supplementary material online, *Tables S14*–*S17*. In summary, dementia risk was U-shaped across systolic pressure in a mid-life AF cohort (*n* = 171 228), with the lowest risk at 120 mmHg and comparable increases above and below that value.^19^

Age modified both associations in the large cohort (*n* = 338 747): the systolic gradient for ischaemic stroke and for all-cause mortality was steepest at 40–49 years and attenuated progressively, in patients aged ≥ 70 years an elevated SBP conferred only a modest excess of stroke and no excess mortality (both *P* for interaction < 0.001); the same attenuation with age was evident for DBP.^21^

In a *post-hoc* analysis pooling CHF and AFFIRM trials (*n* = 5436), the association between SBP and mortality was confined to patients with a left ventricular ejection fraction ≤ 40%, in whom it was U-shaped, and was absent in those with preserved ejection fraction.^32^

Renal function neither predicted mortality after adjustment nor modified the BP associations in a single registry (*n* = 1402).^25^

#### BP TTR and clinical outcomes

Three studies reported SBP-TTR, using different target definitions, referent groups and analytical approaches, and were therefore not pooled (see Supplementary material online, *Tables S10*–*S12*). Kim *et al*. evaluated SBP-TTR below 130 mmHg in 9092 hypertensive patients with AF and found a graded increase in thromboembolic risk as TTR fell: relative to the highest quartile, the adjusted HR for stroke or systemic embolism was 1.33 (1.08–1.63) in the third quartile, 1.43 (1.16–1.75) in the second and 1.55 (1.27–1.89) in the lowest. A consistent pattern was seen for DBP-TTR below 80 mmHg, with HRs of 1.22 (1.01–1.48) and 1.42 (1.16–1.74) in the second lowest and lowest quartiles relative to the combined highest quartiles. The COOL-AF registry examined a target of 120–140 mmHg using the lowest tertile as referent, so its estimates run in the opposite direction: all-cause mortality was lower in the highest TTR tertile (adjusted HR 0.62, 0.48–0.80). Kodani *et al*. examined targets of 110–130 and 120–140 mmHg in the J-RHYTHM registry; each 1% increase in SBP-TTR was associated with a lower incidence of thromboembolism in unadjusted analysis, but this did not persist after adjustment for BP at the time closest to the event, and no categorical comparison reached significance for thromboembolism in either target range.

#### Net clinical benefit of BP control

Two studies explicitly evaluated ischaemic stroke prevention and ICH risk related to OAC across SBP strata.^20,26^ NCB favoured NOAC over warfarin across nearly all SBP range in both studies, and the magnitude of benefit increased at higher SBP, reaching NCB +4.69 per 100 patient-years at SBP 145–165 mmHg in ENGAGE AF-TIMI 48, driven by markedly higher stroke and ICH rates with warfarin. The sole exception was the 125–134 mmHg stratum in this study (NCB −0.24), where a higher reported ICH rate with NOAC marginally favoured warfarin. In an ANAFIE sub-study, NCB favoured NOAC at every stratum, with the greatest benefit at highest SBP (NCB +1.88 at SBP 150–170 mmHg; *Figure 4*; Supplementary material online, *Table S13*).

**Figure 4:**
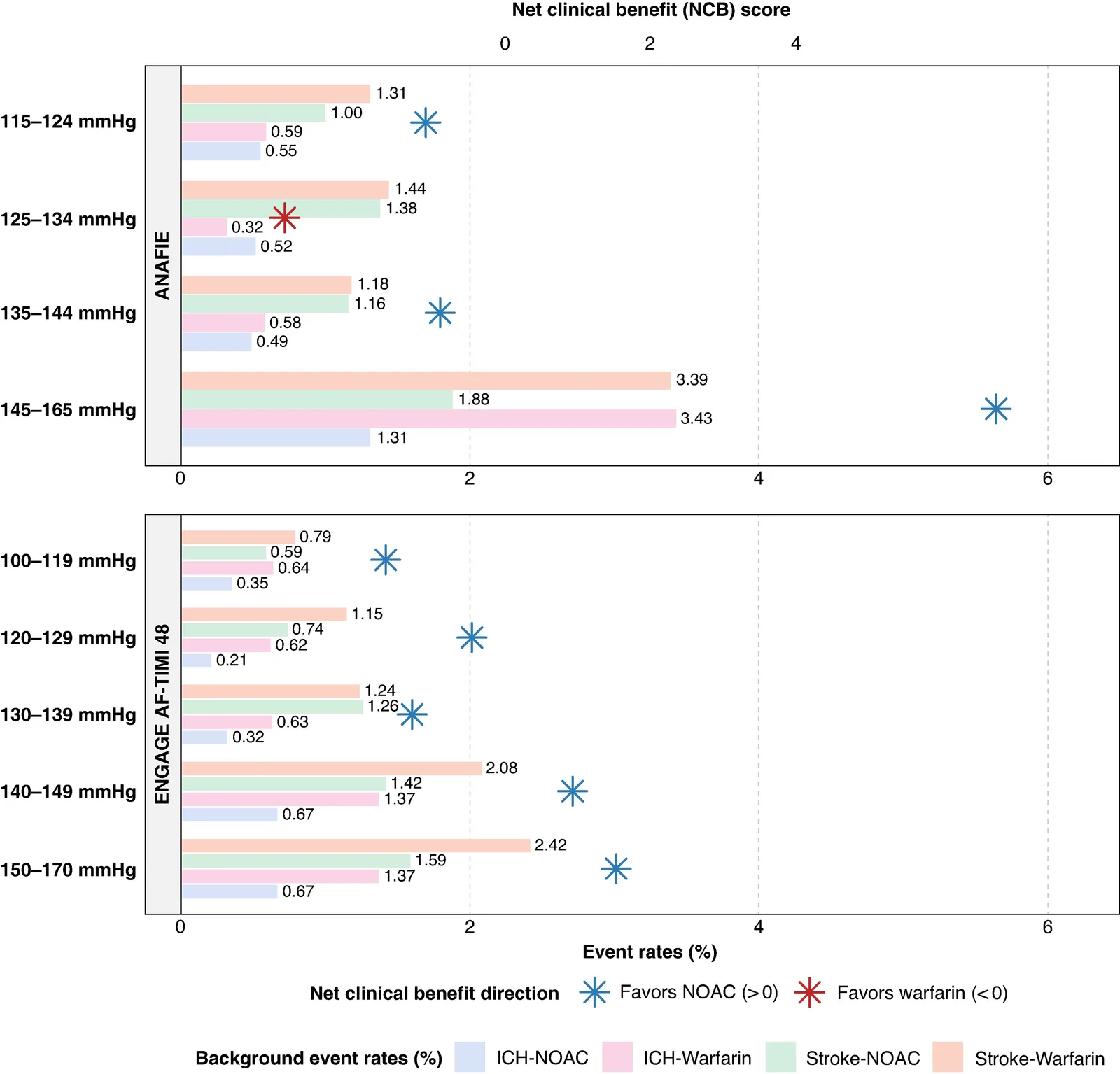
NCB of NOAC vs. warfarin by systolic BP stratum in two contributing studies. NCB is calculated as (Stroke_warfarin_ − Stroke_NOAC_) − 1.5 (ICH_NOAC_ − ICH_warfarin_), where all rates are per 100 patient-years and the coefficient 1.5 reflects the greater clinical disutility of intracranial haemorrhage vs. ischaemic stroke. Coloured horizontal bars represent absolute rates for stroke and ICH by treatment group. Asterisks indicate the NCB direction for each stratum, where blue asterisks (*) denote NCB favouring NOAC (>0) and red asterisks (*) denote NCB favouring warfarin (<0).

NCB was also estimable across diastolic strata in the ENGAGE AF-TIMI 48 analysis. NOAC was favoured at every level: +0.84 per 100 patient-years below 75 mmHg, +0.41 at 75–<85 mmHg, +0.77 at 85–<90 mmHg and +2.67 at ≥90 mmHg with no stratum favouring warfarin.

## Discussion

Our meta-analysis represents the largest and most comprehensive evaluation of optimal BP levels in patients with AF across different clinical outcomes. A U-shaped association was observed for SBP with all-cause mortality, and a J-shaped association was found for DBP with all-cause mortality, with an elevated risk only at lower DBP levels. Positive monotonic trends were observed for SBP with intracranial haemorrhage, ischaemic stroke, and stroke or systemic embolism, with a markedly steeper gradient for intracranial haemorrhage than for ischaemic events. While some of our findings confirm previous observations, they also provide new insights into the complex relationship between BP and outcomes in this population.

Perhaps the most important finding was the consistent U-shaped association between SBP and all-cause mortality. This type of association is also consistent with observations from large cardiovascular cohorts outside AF, including analyses from the ONTARGET/TRANSCEND^35^ and the PROVE-IT/TIMI-22 studies,^36^ which also reported increased mortality at both low and high SBP levels.

In contrast, ischaemic stroke, systemic embolism and intracranial haemorrhage demonstrated predominantly monotonic relationships with SBP, with no clear evidence of excess risk at lower levels. That all-cause mortality should take a different form from its two most visible causes appears paradoxical but is reconcilable. Stroke and intracranial haemorrhage account for only a small fraction of deaths in anticoagulated AF, of the order of 5–7% each in cause-of-death analyses of contemporary anticoagulation trials, whereas progressive heart failure, sudden death and non-cardiovascular causes together account for the large majority.^37^ All-cause mortality is therefore dominated by pathways on which low-BP is a recognized prognostic marker of advanced disease, and only marginally by the cerebrovascular endpoints modelled separately here. Elevated SBP directly contributes to vascular remodelling, endothelial dysfunction and atrial cardiomyopathy, providing a possible explanation for the progressive increase in ischaemic stroke and ICH observed with higher BP. Conversely, low SBP may be a marker of advanced disease rather than a causal factor for death: patients with low-BP carry a greater burden of comorbid conditions such as heart failure and autonomic dysfunction, as well as broader clinical syndromes including frailty and cachexia, each of which contributes substantially to mortality risk. Low SBP may therefore act as an epiphenomenon reflecting severe subclinical disease, terminal decline, or unmeasured frailty not captured by baseline risk-adjustment models. Neither reverse causation nor residual confounding can be excluded from these observational data, and residual confounding may be the more likely of the two: no contributing cohort adjusted for frailty, sarcopenia, weight loss, albumin or functional status, and none reported landmark analyses excluding early deaths. In contrast, renal function did not account for the trend: eGFR was associated with mortality in univariable but not multivariable analyses, and no interaction was observed with either SBP or DBP category.

The observation that low systolic pressure marks frailty and advanced comorbidity rather than causing harm is also supported by the interaction between age and BP (see Supplementary material online, *Table S13*).^18^ Notably, in patients aged ≥ 70 years, an SBP of 120–140 mmHg was associated with lower mortality than an SBP below 100 mmHg, while an SBP ≥ 160 mmHg was not associated with excess mortality in this age group. This is consistent with the U-shape observed in our pooled analysis and with the interpretation that very low-BP in older patients with AF marks frailty and advanced disease rather than conferring benefit. Together with the attenuation of the BP–stroke gradient with age, these observations argue against applying a single target across the age spectrum, and support more permissive targets in older patients, a position consistent with the 2024 ESC guideline recommendation of 140 mmHg or ‘as low as reasonably achievable’ in patients who are frail or aged over 85 years.^8^ Although exploratory, these findings are biologically plausible given the greater burden of competing risks in older individuals and support a more individualized approach to BP targets in elderly AF patients. A similar interaction was observed according to left ventricular ejection fraction. In a *post-hoc* analysis, the association between SBP and mortality was evident only in patients with EF <40% and not in those with preserved EF.^32^ This finding supports the notion that low SBP may act primarily as a marker of advanced cardiovascular disease rather than a direct cause of increased mortality.

These findings describe an older, predominantly anticoagulated population with largely prevalent AF receiving OAC. Their transferability to younger patients, to those not anticoagulated, and to newly diagnosed AF is uncertain and, in the case of age, appears limited. In the one cohort reporting age-stratified estimates, the systolic gradients for both ischaemic stroke and mortality were steepest at 40–49 years and attenuated progressively with age (both *P* for interaction <0.001). The low-pressure mortality signal that underlies our caution regarding intensive targets is therefore substantially a phenomenon of older patients with a high burden of competing risk and provides little justification for permissive targets in younger individuals, in whom the ischaemic gradient is steepest and conventional hypertension targets are more likely to apply.

Taken together, these findings are compatible with a systolic range of ∼120 to 130 mmHg in patients with AF. This range is not an empirically observed optimum for any single endpoint and is instead derived from the intersection of divergent risk gradients, in which ischaemic and haemorrhagic events rise progressively above 120 mmHg while mortality rises below ∼125 mmHg. No single value minimized all outcomes simultaneously, and the choice of where within this region to sit therefore depends on which endpoint is prioritized for an individual patient. Because these estimates derive from observational cohorts and cannot exclude reverse causation or residual confounding, this range should be regarded as hypothesis-generating and as a basis for trial design rather than as a treatment recommendation.

Findings relating to DBP are also noteworthy. In fact, DBP demonstrated a J-shaped association with mortality, with risk rising as pressure fell that mirror image the pattern observed for SBP. Excess risk became statistically significant below 76 mmHg, reaching 1.49 (1.15–1.92) at 55 mmHg. A similar pattern was evident in individual cohorts: in RE-LY,^15^ baseline DBP below 70 mmHg was associated with a 37% higher mortality relative to 70–79 mmHg, and in the ENGAGE AF-TIMI 48 trial,^26^ average DBP below 65 mmHg was associated with a 78% higher risk of major bleeding. Several mechanisms may explain this observation. Coronary perfusion occurs predominantly during diastole, and excessively low DBP may impair myocardial blood flow. In addition, similarly to low SBP, low DBP may represent a surrogate marker of frailty, heart failure, reduced stroke volume, or advanced vascular disease. Taken together, these data support avoiding DBP below ∼75–80 mmHg in patients with AF. We are unable to define an upper bound as no estimate above the referent differed significantly from unity, and few participants contributed data above 100 mmHg.

The 2024 ESC guidelines on elevated BP and hypertension recommend a systolic target of 120–129 mmHg for adults receiving BP-lowering medication, with an opt-out for intolerance, orthostatic symptoms, frailty, age over 85 years, or limited life expectancy, and identify 120 mmHg as the optimal point within that range.^38^ The 2024 ESC guidelines for AF adopt the same default target of 120–129/70–79 mmHg in most adults, with 140 mmHg or a pressure ‘as low as reasonably achievable’ in patients who are clinically frail or aged over 85 years.^8^ Our estimates are broadly compatible with the upper boundary of these recommendations but sit less comfortably with their lower boundary. The nadir of SBP for all-cause mortality was 134 mmHg with significantly lower mortality at 130 mmHg than at the 120 mmHg referent (RR 0.93; 0.88–0.98), whereas at 110 mmHg it was significantly higher (RR 1.14; 1.05–1.24). The ESC position that 120 mmHg is the optimal point within the target range is therefore not supported by these data in patients with AF. This divergence is consistent with the exclusion of AF patients from the trials underpinning intensive targets, most notably SPRINT.^39^ However, it should not be read as evidence against intensive treatment: our data are observational, cannot exclude reverse causation, and the guideline recommendations rest on randomized evidence that does not yet exist for AF. It does support the guidelines' own emphasis on tolerability and individualization and argues that the lower boundary in particular warrants dedicated trial evaluation before intensive targets are applied uniformly in this population.

Beyond absolute BP values, growing evidence suggests that BP stability may also be important. Across the available studies, patients who spent a greater proportion of time within pre-defined target ranges experienced lower rates of all-cause mortality. A related literature has examined visit-to-visit variability directly.^40–42^ In a sub analysis of AFFIRM,^41^ the highest quartile of systolic variability was independently associated with stroke, major bleeding and all-cause death. Variability was also inversely related to TTR in that cohort, suggesting the two phenomena are linked rather than independent. These findings reinforce the concept that sustained BP control may provide benefits beyond values captured by single clinic measurements.

Finally, our NCB analysis has important therapeutic implications. NOAC therapy demonstrated superior NCB compared to warfarin across virtually all SBP strata, and this advantage increased progressively with higher BP levels. The greatest benefit was observed among patients with uncontrolled hypertension, where both thromboembolic and intracranial haemorrhage risks are amplified. Since hypertension is a major determinant of both ischaemic and bleeding events, the superior safety profile of NOACs becomes increasingly important as BP rises. These estimates are nonetheless exploratory. They are best read as consistent with existing guideline preference for NOACs rather than as independent evidence for it, and should not be generalized beyond the populations and pressures sampled here.

### Clinical implications

Our findings suggest that BP has an important influence on several clinical outcomes in individuals with AF. While all-cause mortality follows a U-shaped association with SBP, reflecting the adverse prognostic significance of both uncontrolled hypertension and low-BP as a marker of advanced disease, thromboembolic and haemorrhagic outcomes increase progressively with rising SBP. These findings, paired with the evidence of a positive monotonic association with stroke or ICH, support a possible pragmatic target SBP of 120–130 mmHg in most patients with AF, balancing stroke prevention, bleeding risk, and overall survival. However, the weaker associations observed in elderly patients and those with preserved ventricular function suggest that this approach may not be appropriate across all categories and that BP management should remain individualized. For DBP, the data suggest a lower safety threshold around 75–80 mmHg, below which risk increases. Conversely, no upper risk threshold could be established because estimates above 80 mmHg did not differ significantly from the reference group due to limited statistical power. Finally, the superior NCB of NOACs across all BP strata, particularly in patients with uncontrolled hypertension, reinforces current recommendations favouring NOACs over warfarin and highlights the importance of integrating optimal BP control with anticoagulation strategies.

### Limitations

Several limitations apply. Between-study heterogeneity was substantial, particularly for all-cause mortality, although the U-shaped association persisted in every leave-one-out model. As a study-level synthesis it cannot exclude genuine effect modification across populations, and subgroup analyses by sex, age and AF type were not feasible; individual-participant data, permitting harmonized adjustment and joint modelling of the two pressure components, would be required to resolve these questions. Because no study reported outcomes cross-classified by systolic and diastolic strata, the two associations are modelled marginally rather than jointly, and most cohorts contributed a single baseline measurement that does not capture variability over time. Cohorts were recruited between 2001 and 2023, over which recommended targets fell substantially, so an identical pressure may reflect untreated hypertension in an earlier cohort and treated hypertension in a later one. Observational designs carry an inherent risk of residual confounding and reverse causality, particularly at low pressures in patients with advanced comorbidity or unmeasured frailty. Cardiovascular death, dementia, renal function, effect modification by age and ejection fraction, and TTR were each informed by one or two cohorts and reported descriptively. No diastolic estimate above the referent differed from unity, but few participants contributed data above 100 mmHg; this reflects imprecision at the upper extreme rather than a true plateau. The NCB analysis is exploratory: two studies differing in design, agent, measurement method and strata, described rather than pooled, and dependent on a fixed weighting for intracranial haemorrhage.

## Conclusions

In this large dose-response meta-analysis of AF patients, all-cause mortality followed a U-shaped association with systolic pressure, with a nadir at ∼134 mmHg, and a J-shaped association with diastolic pressure, with a nadir at 83 mmHg. Ischaemic stroke, intracranial haemorrhage, and stroke/systemic embolism showed consistent monotonic increases with rising SBP. These findings support a pragmatic target of SBP 120–130 mmHg as the range offering the most favourable balance between thromboembolic, haemorrhagic, and mortality risk in patients with AF, recognising that haemorrhagic risk is already rising at the upper end of this range. A more individualized approach should be considered in elderly patients and those with reduced ventricular function, in whom these associations appear attenuated. This synthesis is derived from observational data, causal inference is not possible, and the proposed range should be treated as hypothesis-generating. Randomized trials specifically targeting BP in AF populations are needed to confirm these observations.

## Supplementary Material

euag223_Supplementary_Data

## Data Availability

This being a systematic review and meta-analysis does not have any original data that is not already available in prior publications. Reasonable requests to collaborate with the data can be addressed via the corresponding author.
