# Supplementary material for: Non-linear associations between blood pressure and clinical outcomes in atrial fibrillation: a systematic review and dose-response meta-analysis
Source: Europace. 2026 Aug 28;28(10):euag223. doi: 10.1093/europace/euag223 (PMC13660803; doi:10.1093/europace/euag223)
Supplement: Supplementary file 1 [file euag223_supplementary_data.pdf]

Supplementary Appendix

This appendix has been provided by the authors to give readers additional information about their work. Supplement to: Shahmohamadi E, et al. Non-linear associations between blood pressure and clinical outcomes in atrial fibrillation: A systematic review and dose-response meta-analysis

Contents

Supplemental Methods 1: PRISMA checklist ... 3
Supplemental Methods 2: MOOSE checklist ... 6
Supplemental Methods 3. PECOS Framework ... 9
Supplemental Methods 4. Search details ... 11
Supplemental Methods 5. Dose-response modelling ... 12
Supplementary Table S1. Blood pressure measurement methods by cohort ... 13
Supplementary Table S2. Outcome definitions and model adjustments by cohort ... 15
Supplementary Table S3. Contribution of each cohort to each analysis ... 17
Supplementary Table S4. Analyses by exposure and outcome, with contributing cohorts ... 19
Figure S1. Assessment of risk of bias using the ROBINS-E risk of tool for non-randomised observational cohorts ... 20
Figure S2. Assessment of risk of bias using the ROB-2 risk of tool for randomised observational cohorts ... 21
Supplementary Table S5. Model characteristics and fitted curve shape, systolic blood pressure. ... 22
Supplementary Table S6. Pooled relative risk per 10 mm Hg increase in systolic blood pressure, with heterogeneity. ... 23
Supplementary Figure S3. Leave-one-out sensitivity analysis for All-cause mortality associated with systolic blood pressure ... 24
Supplementary Figure S4. Funnel plots ... 25
Supplementary Figure S5. Sensitivity Analysis Restricting to Studies Reporting Hazard Ratios ... 26
Supplementary Table S7. Pooled Hazard Ratios (95% CI) at Key SBP Thresholds: Sensitivity Analysis Restricted to Hazard Ratio Studies ... 27
Supplementary Table S8. Pooled relative risk (95% CI) for all-cause mortality at key diastolic blood pressure thresholds, referent 80 mm Hg ... 28
Supplementary Table S9. Cohorts contributing diastolic blood pressure data, by outcome ... 29
Supplementary Table S10. Study characteristics of cohorts contributing to systolic blood pressure time-in-target-range analyses ... 30
Supplementary Table S11. SSE / thromboembolic outcomes by SBP-TTR group ... 31
Supplementary Table S12. All-cause and cardiovascular mortality outcomes by SBP-TTR group ... 32
Supplementary Table S13. Intracranial haemorrhage and Ischemic stroke, stratified by blood pressure control , and calculated net clinical benefit ... 33
Supplementary Results ... 35
Supplementary Table S14. Blood pressure and incident dementia in a single cohort ... 36
Supplementary Table S15. Age-stratified associations of blood pressure with ischaemic stroke and all-cause mortality in a single cohort ... 37
Supplementary Table S16. Effect modification of the systolic blood pressure–mortality association by left ventricular ejection fraction in a single post-hoc analysis ... 38

Supplementary Table S17. Renal function, mortality and blood pressure in a single registry 39
Supplementary Table S18. Exploratory subgroup analyses and meta-regression for the systolic blood pressure and mortality association 40

Supplemental Methods 1: PRISMA checklist

Section/topic	Item No	Checklist item	Reported on page No
**Title**			
Title	1	Identify the report as a systematic review, meta-analysis, or both	1
**Abstract**			
Structured summary	2	Provide a structured summary including, as applicable, background, objectives, data sources, study eligibility criteria, participants, interventions, study appraisal and synthesis methods, results, limitations, conclusions and implications of key findings, systematic review registration number	2
**Introduction**			
Rationale	3	Describe the rationale for the review in the context of what is already known	Introduction
Objectives	4	Provide an explicit statement of questions being addressed with reference to participants, interventions, comparisons, outcomes, and study design (PICOS)	Introduction
**Methods**			
Protocol and registration	5	Indicate if a review protocol exists, if and where it can be accessed (such as web address), and, if available, provide registration information including registration number	Methods
Eligibility criteria	6	Specify study characteristics (such as PICOS, length of follow-up) and report characteristics (such as years considered, language, publication status) used as criteria for eligibility, giving rationale	Methods
Information sources	7	Describe all information sources (such as databases with dates of coverage, contact with study authors to identify additional studies) in the search and date last searched	Methods
Search	8	Present full electronic search strategy for at least one database, including any limits used, such that it could be repeated	Appendix 3
Study selection	9	State the process for selecting studies (that is, screening, eligibility, included in systematic review, and, if applicable, included in the meta-analysis)	Methods
Data collection process	10	Describe method of data extraction from reports (such as piloted forms, independently, in duplicate) and any processes for obtaining and confirming data from investigators	Methods
Data items	11	List and define all variables for which data were sought (such as PICOS, funding sources) and any assumptions and simplifications made	Methods
Risk of bias in individual studies	12	Describe methods used for assessing risk of bias of individual studies (including specification of whether this was done at the study or outcome level), and how this information is to be used in any data synthesis	Methods

Section/topic	Item No	Checklist item	Reported on page No
Summary measures	13	State the principal summary measures (such as risk ratio, difference in means).	Methods
Synthesis of results	14	Describe the methods of handling data and combining results of studies, if done, including measures of consistency (such as I^2 statistic) for each meta-analysis	Methods
Risk of bias across studies	15	Specify any assessment of risk of bias that may affect the cumulative evidence (such as publication bias, selective reporting within studies)	Methods
Additional analyses	16	Describe methods of additional analyses (such as sensitivity or subgroup analyses, meta-regression), if done, indicating which were pre-specified	Methods
**Results**			
Study selection	17	Give numbers of studies screened, assessed for eligibility, and included in the review, with reasons for exclusions at each stage, ideally with a flow diagram	Results, Figure 1
Study characteristics	18	For each study, present characteristics for which data were extracted (such as study size, PICOS, follow-up period) and provide the citations	Results, Table 1
Risk of bias within studies	19	Present data on risk of bias of each study and, if available, any outcome-level assessment (see item 12).	Results, Appendix 4
Results of individual studies	20	For all outcomes considered (benefits or harms), present for each study (a) simple summary data for each intervention group and (b) effect estimates and confidence intervals, ideally with a forest plot	
Synthesis of results	21	Present results of each meta-analysis done, including confidence intervals and measures of consistency	Results, Figures 2-5; Appendices 5-14
Risk of bias across studies	22	Present results of any assessment of risk of bias across studies (see item 15)	Not applicable
Additional analysis	23	Give results of additional analyses, if done (such as sensitivity or subgroup analyses, meta-regression) (see item 16)	Not applicable
**Discussion**			
Summary of evidence	24	Summarise the main findings including the strength of evidence for each main outcome; consider their relevance to key groups (such as health care providers, users, and policy makers)	Discussion
Limitations	25	Discuss limitations at study and outcome level (such as risk of bias), and at review level (such as incomplete retrieval of identified research, reporting bias)	Discussion
Conclusions	26	Provide a general interpretation of the results in the context of other evidence, and implications for future research	Discussion
**Funding**			

Section/topic	Item No	Checklist item	Reported on page No
Funding	27	Describe sources of funding for the systematic review and other support (such as supply of data) and role of funders for the systematic review	After Discussion

Supplemental Methods 2: MOOSE checklist

Criteria		Brief description of how the criteria were handled in the review
**Reporting of background**		
√	Problem definition	What are the optimal blood pressure (BP) targets for patients with atrial fibrillation (AF)?
√	Hypothesis statement	Both elevated and low BP confer excess risk of adverse outcomes (ischaemic stroke, intracranial haemorrhage, and death) in patients with AF; i.e. a non-linear, U/J-shaped exposure–outcome relationship , and uncontrolled hypertension modifies the net clinical benefit of oral anticoagulation.
√	Description of study outcomes	Primary: all-cause mortality Secondary: stroke or systemic thromboembolism (ischaemic stroke or systemic embolism), cardiovascular mortality, intracranial haemorrhage, major bleeding, heart failure, AF progression/recurrence, and dementia/cognitive decline. Derived: net clinical benefit (NCB) of each anticoagulant strategy per BP stratum
√	Type of exposure	Blood pressure : systolic (SBP) and diastolic (DBP) blood pressure, and systolic BP time in target range (TTR).
√	Type of study designs used	Randomised controlled trials and their post-hoc analyses, prospective and retrospective cohort studies, and registry analyses.
√	Study population	Adults (≥ 18 years) with established AF of any type (paroxysmal, persistent, long-standing persistent, or permanent).
**Reporting of search strategy should include**		
√	Qualifications of searchers	Elnaz Shahmohamadi, MD, MPH; Alessandra Pina, MD
√	Search strategy, including time period included in the synthesis and keywords	Time period: from database inception to May 2026. MeSH/Emtree controlled vocabulary and free-text terms covering (i) atrial fibrillation, (ii) blood pressure and hypertension, and (iii) the pre-specified outcomes. The full strategy is provided in **Supplementary File 1.**
√	Databases and registries searched	MEDLINE, Embase, and Scopus. Prospectively registered on PROSPERO (ID CRD420251128571).
√	Search software used, name and version, including special features	OvidSP (Embase and MEDLINE searching); Covidence used to manage references
√	Use of hand searching	We searched bibliographies of retrieved papers
√	List of citations located and those excluded, including justifications	Documented in the PRISMA 2020 flow diagram; reasons for exclusion were reported in **Supplementary**
√	Method of addressing articles published in languages other than English	Not applicable
√	Method of handling abstracts and unpublished studies	Excluded

√	Description of any contact with authors	None
**Reporting of methods should include**		
√	Description of relevance or appropriateness of studies assembled for assessing the hypothesis to be tested	Pre-specified inclusion and exclusion criteria are described in the Methods section.
√	Rationale for the selection and coding of data	Data were extracted independently by two reviewers using a piloted form, covering study and population characteristics, design, BP exposure (strata, levels, and TTR), anticoagulation, and outcomes.
√	Assessment of confounding	Adjusted effect estimates were extracted preferentially; confounding was incorporated into the risk-of-bias assessment.
√	Assessment of study quality, including blinding of quality assessors; stratification or regression on possible predictors of study results	The Cochrane Risk of Bias in Non-randomised Studies – of Interventions (ROBINS-I) tool was used to assess the risk of bias within individual observational studies. The Cochrane RoB 2 tool (RCTs and post-hoc RCT analyses) was applied by two independent reviewers
√	Assessment of heterogeneity	Quantified with Cochran's Q and the I^2 statistic and explored through pre-specified subgroup and sensitivity analyses where data permitted.
√	Description of statistical methods in sufficient detail to be replicated	Description of methods of meta-analyses, sensitivity analyses, meta-regression and assessment of publication bias are detailed in the methods. Analyses were performed in R 4.3.0 (meta, metafor, dosresmeta, rms).
√	Provision of appropriate tables and graphics	Table 1; Figures 1-4; Appendices tables 4-6
**Reporting of results should include**		
√	Graph summarizing individual study estimates and overall estimate	Appendices Tables 2-4
√	Table giving descriptive information for each study included	Table 1 (study and population characteristics).
√	Results of sensitivity testing	Leave-one-out influence analysis.
√	Indication of statistical uncertainty of findings	95% confidence intervals are reported for all summary estimates, alongside I^2 values and tests for non-linearity.
**Reporting of discussion should include**		
√	Quantitative assessment of bias	Small-study effects and publication bias were assessed (funnel plots, Egger's and Begg's tests) for outcomes with ≥ 10 contributing studies; limitations are discussed.
√	Justification for exclusion	All studies were excluded based on the pre-defined inclusion criteria in methods section.
√	Assessment of quality of included studies	Summarised through the risk-of-bias tools , discussion included in 'Methods' section

**Reporting of conclusions should include**		
√	Consideration of alternative explanations for observed results	Discussion
√	Generalization of the conclusions	Discussed in the context of the included populations (predominantly anticoagulated AF cohorts, registries, and trials).
√	Guidelines for future research	Randomised trials of blood-pressure targets specifically in patients with AF are recommended.
√	Disclosure of funding source	In "Acknowledgement" section

Supplemental Methods 3. PECOS Framework

Element	Inclusion Criteria	Exclusion Criteria
**Population**	• Adults aged ≥ 18 years with established atrial fibrillation of any type (paroxysmal, persistent, long-standing persistent or permanent), confirmed electrocardiographically or by equivalent clinical documentation.<br>• Any care setting; anticoagulated and non-anticoagulated cohorts eligible.	• populations without documented AF, and cohorts in which AF status could not be separated from the wider population.
**Exposure**	• Systolic and diastolic blood pressure, reported as categorical strata or as a continuous variable.<br>• Time in target range (TTR) extracted where reported and analysed descriptively.	• Studies reporting only hypertension status as a binary variable were excluded, as no dose could be assigned.
**Comparator**	• Within-study reference stratum, re-expressed to a common referent of 120 mm Hg systolic and 80 mm Hg diastolic at the prediction stage.<br>• For the net clinical benefit analysis: non-vitamin K antagonist oral anticoagulant versus warfarin within each blood-pressure stratum.	• Excluded where the reference category was defined by hypertension status rather than by a blood-pressure level, or by a joint systolic-and-diastolic range, since no dose could be assigned to the referent.
**Outcomes**	**Primary**<br>• All-cause mortality.<br>**Secondary**<br>• Ischaemic stroke<br>• Intracranial haemorrhage<br>• Stroke or systemic embolism (composite)<br>• Major bleeding (ISTH definition or study equivalent)<br>• Cardiovascular death<br>• Dementia or cognitive decline<br>**Derived**<br>• Net clinical benefit per blood-pressure stratum, calculated by the Singer–Lip formula with a weighting of 1.5 for intracranial haemorrhage relative to ischaemic stroke.	No information on an BP outcome

Element	Inclusion Criteria	Exclusion Criteria
**Study design**	• Randomised controlled trials and their pre-specified or post-hoc blood-pressure subgroup analyses; prospective and retrospective cohort studies; case-control studies.<br>• No language or date restrictions.<br>• Where several reports derived from one source population, each was included only if it addressed a distinct outcome; no outcome pooled more than one such report.	• case reports, editorials, letters, narrative reviews, and studies with fewer than two blood-pressure strata.

Supplemental Methods 4. Search details

	Query	Results (19 May 2026)
	**PubMed**	
#1	(“atrial fibrillation”[Title/Abstract] OR "AF"[Title/Abstract] OR "Atrial Fibrillation"[Mesh])	*83,671*
#2	("Systolic Blood Pressure"[tiab] OR "SBP"[tiab] OR "Blood Pressure Variability"[tiab] OR "blood pressure target"[tiab])	*74,383*
#3	("Mortality"[Mesh] OR "Death"[tiab] OR "Stroke"[Mesh] OR "Ischemic Stroke"[tiab] OR "Hemorrhage"[Mesh] OR "Bleeding"[tiab] OR "Hospitalization"[Mesh] OR "Hospitalisation"[tiab] OR "Outcomes"[tiab] OR "Prognosis"[tiab] OR "Dementia"[tiab] OR "Recurrence"[tiab]))	*4,964,836*
#4	#1 AND #2 AND #3	***1527***
	**SCOPUS**	
#1	(TITLE-ABS-KEY("atrial fibrillation") OR TITLE-ABS-KEY("AF"))	*103,622*
#2	(TITLE-ABS-KEY("systolic blood pressure" OR "sbp" OR "blood pressure variability" OR "blood pressure target")	*252,340*
#3	(TITLE-ABS-KEY("mortality" OR "death" OR "stroke" OR "bleeding" OR "hemorrhage" OR "hospitalization" OR "hospitalisation" OR "outcomes" OR "dementia" OR "recurrence"))	*10,265,111*
#4	#1 AND #2 AND #3	***723***
	**Embase**	
#1	(“atrial fibrillation”:ti,ab,kw OR “AF”:ti,ab,kw)	*22,912*
#2	('systolic blood pressure'/exp OR 'systolic blood pressure':ti,ab OR 'sbp':ti,ab OR 'blood pressure variability':ti,ab OR 'blood pressure target:ti,ab)	
#3	('mortality'/exp OR 'death':ti,ab OR 'stroke'/exp OR 'ischemic stroke':ti,ab OR 'bleeding'/exp OR 'hemorrhage':ti,ab OR 'hospitalization'/exp OR 'hospital admission':ti,ab OR 'prognosis'/exp OR 'dementia'/exp OR 'recurrence'/exp)	*3,112,836*
#4	#1 AND #2 AND #3	***1109***
	**Cochrane Library**	
#1	([mh "Atrial Fibrillation"] OR "atrial fibrillation":ti,ab,kw OR "af":ti,ab,kw)	*16,821*
#2	([mh "Systolic Blood Pressure"] OR [mh "Blood Pressure"] OR "systolic blood pressure":ti,ab,kw OR "sbp":ti,ab,kw OR "blood pressure variability":ti,ab,kw OR "blood pressure target":ti,ab,kw)	*23,864*
#3	([mh "Mortality"] OR [mh "Stroke"] OR [mh "Hemorrhage"] OR [mh "Hospitalization"] OR "death":ti,ab,kw OR "stroke":ti,ab,kw OR "ischemic stroke":ti,ab,kw OR "bleeding":ti,ab,kw OR "hospitalization":ti,ab,kw OR "hospitalisation":ti,ab,kw OR "outcomes":ti,ab,kw OR "prognosis":ti,ab,kw))	*53,278*
#4	#1 AND #2 AND #3	***459***
**Citation searching**		***20***
**TOTAL**		***3928***
**TOTAL AFTER REMOVING DUPLICATES**		***2784***

Supplemental Methods 5. Dose-response modelling

Within-study covariance

Estimates within a study are correlated because all strata share a common referent. Where the number of events and participants was reported for every stratum, this correlation was approximated by the method of Greenland and Longnecker.[1] Where per-stratum event counts were not reported, the published variances were supplied directly and an independent within-study covariance structure was assumed. This applied to all-cause mortality, stroke or systemic embolism, and major bleeding for systolic pressure, and to all-cause mortality for diastolic pressure. For one cohort (Lip et al., SPORTIF III/V) reporting relative risks without confidence intervals, variances were derived from the number of events and participants in each stratum.

Exposure assignment

Studies reporting categorical strata contributed the stratum midpoint or, where available, the reported mean pressure within the stratum. For open-ended upper and lower categories, the interval width was estimated as 1.25 times the width of the adjacent closed interval, and the midpoint assigned accordingly. Strata falling outside 90–200 mm Hg systolic or 50–110 mm Hg diastolic were excluded, as data density outside these ranges was insufficient to support stable spline estimation.

Knot placement

Three knots were placed at the 10th, 50th and 90th percentiles of the exposure distribution, weighted by the number of participants contributing to each stratum, following Harrell's recommendation for three-knot specifications. Both tails were constrained to linearity. Knot positions were prespecified and were held at full-model values in all leave-one-out analyses so that curves remained comparable across models.

Linear dose-response analysis

Studies reporting a continuous per-unit estimate contributed it directly after rescaling to the common increment (10 mm Hg systolic, 5 mm Hg diastolic) on the log scale. Between-study variance was estimated by restricted maximum likelihood; the DerSimonian–Laird estimator was applied as a sensitivity analysis and did not materially alter any pooled estimate.

Sensitivity analysis

To address the heterogeneity of effect measures across included studies, a pre-specified sensitivity analysis was conducted restricting the dose-response models to studies reporting multivariable-adjusted hazard ratios from Cox proportional hazards models.

Software. R version 4.3.0 (*dosresmeta*, *metafor*, *ggplot2*, *dplyr*).

The net clinical benefit (NCB) of NOAC versus warfarin at each BP stratum was calculated using the Singer–Lip formula:

NCB = (Strokewarfarin − StrokeNOAC) − 1.5×(ICHNOAC − ICHwarfarin),

where rates are expressed per 100 patient-years and the coefficient 1.5 reflects the relative disutility of intracranial haemorrhage versus ischaemic stroke, consistent with Singer et al. A positive NCB indicates net benefit of NOAC over warfarin; a negative value indicates net harm. NCB estimates are presented descriptively across BP strata for each contributing study.

Supplementary Table S1. Blood pressure measurement methods by cohort

Cohort	Type of BP Measurement	Time of BP Measurement	BP Assessment Methods
**AFFIRM trial cohort**	Updated systolic BP and diastolic BP	BP measurements were taken post-AF diagnosis	Electronic health record measurements from general practice; the study used the average of available BP values, with reasonable-range checks applied
**SPCCD-SKA**	Baseline systolic BP	Baseline, measured closest to OAC initiation, up to 24 months before index date	Extracted from primary care electronic medical records; baseline SBP defined as the closest available value before index date, with repeated clinical data captured in the database
**RE-LY Trial**	SBP, DBP, Pulse Pressure	Baseline and serial visits (q3–4 mo)	Automatic validated device (Omron HEM 757); sitting; 3 min rest; attended.
**COOL-AF**	SBP-TTR	Baseline and serial 6-month visits	Encouraged 3 measurements and recorded the average.
**F-CREATE**	SBP	Baseline health examination	Mercury, automated, or aneroid; twice measured; mean used.
**J-RISK AF**	SBP	Baseline (enrollment)	Auscultatory or automated; standard clinical practice.
**Korea NHIS**	SBP, DBP	Baseline health screening	Automatic oscillometric or mercury sphygmomanometer; rest >5 min.
**ANAFIE**	Home SBP	Twice daily (morning/evening) for 7 days	Brachial cuff oscillometric method; average of 1 week's measurements.
**Asan Medical**	SBP, DBP	Baeline and serial follow-up (median 14x)	Automatic oscillometric device; average of last 2 of 3 measurements.
**AF-GEN-UK**	SBP, DBP	Baseline routinely collected	Clinicians followed official UK NICE recommendations.
**MIMIC-III**	SBP, DBP, MBP	Hospital admission BP	First record after entering the ICU ward.
**ORBIT-AF**	SBP, DBP	Baseline and serial 6-month visits	Nonstandardized; obtained at enrolling sites per clinical routine.
**SPORTIF III & V**	SBP, DBP	Baseline, 1, 3, 6 mo, then every 6 mo	Average of ~6.5 measurements during the study
**ENGAGE AF-TIMI 48**	SBP, DBP	Serial measurements during trial	Average SBP/DBP before event; post-event values censored
**CPRD**	SBP	Post-DOAC initiation	Closest measurement to DOAC initiation in primary care

**J-RHYTHM**	SBP-TTR	Serial (median 15 readings)	Auscultatory or automated; target 110–130 or 120–140 mmHg
**Chinese AF registry**	SBP, DBP	Admission BP	Brachial BP; average of at least two electronic measurements
**AFFIRM and AF-CHF**	Baseline SBP (single measurement)	At randomisation; not systematically collected thereafter	Measured after the patient had been seated quietly for 5 minutes, in keeping with American Heart Association recommendations.
**Rocket-AF**	Screening SBP and DBP (single measurement)	Screening visit, within 30 days of randomisation	Measured by study personnel with a sphygmomanometer after 5 minutes semi-recumbent. Site devices were not standardised. Study-exit BP was also recorded but was not used, as it generally post-dated the outcomes of interest; interval measurements were not protocolised and were therefore not analysed.

Supplementary Table S2. Outcome definitions and model adjustments by cohort

Author, year	Outcome Definition	Statistical Adjustments / Covariates	Effect measure
**AFFIRM trial cohort**	all-cause mortality, Stroke/SEE, Major bleeding	Sex, age, BMI, smoking status, Indigenous status, socioeconomic status, education level, CHA2DS2-VASc score, multimorbidity, BP-lowering medication use, GP visits, regularity, and continuity of care; random effects for GP clinic	OR
**SPCCD-SKA**	Primary outcome: haemorrhagic stroke secondary outcomes: any stroke, ischaemic stroke, any bleed, gastrointestinal bleed, all-cause death	Age, sex, high alcohol intake, platelet-inhibition, cholesterol, cerebrovascular disease, diabetes	HR
**RE-LY Trial**	All-cause death, stroke/SEE, major bleeding, any bleeding.	Age, BMI, CrCl, gender, AF type, HF, HTN, prior stroke, CAD, MI, diabetes, valvular disease, ethnicity, OAC, medications.	HR
**COOL-AF**	Ischemic stroke/TIA, ICH, all-cause death, major bleeding, heart failure.	Age, sex, AF type, HF, CAD, CIED, IS/TIA, diabetes, HTN, smoking, dyslipidemia, RRT, dementia, bleeding, medications	HR
**F-CREATE**	Cardiovascular disease (stroke, MI composite), intracranial bleeding, extracranial bleeding.	Age, sex, BMI, smoking, daily drinking, regular exercise, diabetes, HDL, LDL, BP/lipid lowering medications	HR
**J-RISK AF**	Ischaemic stroke, major bleeding, all-cause death, cardiovascular death.	Model 2: CHA2DS2-VASc components, AF type, cardiomyopathy, HTN drugs, BMI, eGFR, haemoglobin	HR
**Korea NHIS**	Ischemic stroke, major cardiovascular events, all-cause death.	Age, sex, BMI, smoking, alcohol, exercise, comorbidities (HF, stroke, MI, PAD, CKD, etc.), medications, CHA2DS2-VASc	HR
**ANAFIE**	Net CV outcome (stroke/SEE + major bleed), ICH, all-cause death.	Age, BMI, major bleed hx, AF type, diabetes, heart disease, cerebrovascular disease, cancer, dementia, falls, medications	HR
**Asan Medical**	Ischemic stroke and systemic embolism.	Age, gender, diabetes, vascular disease, previous stroke, heart failure, use of oral anticoagulants	HR
**AF-GEN-UK**	All-cause death, major bleeding, thromboembolic events (stroke, TIA, PE, DVT).	Age, oral anticoagulation use, CHA2DS2-VASc score, estimated baseline eGFR	HR
**MIMIC-III**	Hospital, 7-day, 30-day, and 1-year mortality.	Age, sex, CHF, valvular disease, stroke, CKD, chronic bronchitis, depression, diabetes, malignancy	OR[b]

**ORBIT-AF**	Stroke/TIA/SE, MI, major bleeding, CV hospitalization, mortality.	Statin and antiplatelet use, randomized treatment, and selected validated risk factors (e.g., age, heart failure, prior stroke)	HR
**SPORTIF III & V**	Stroke and systemic embolic events (primary); mortality; major bleeding	None	RR[a]
**ENGAGE AF-TIMI 48**	Stroke or systemic embolic event (primary); ISTH major bleeding (safety)	Age, sex, CHADS2, CrCl, body weight, meds, stroke history, HF, DM, AF type, smoking, region	HR
**CPRD**	Stroke, MI, intracranial bleed, GI bleed, or death	Age, sex, alcohol, history of stroke/IHD, dementia, liver disease	HR
**J-RHYTHM**	Thromboembolism, major hemorrhage, all-cause death, cardiovascular death	Model 2: CHA2DS2-VASc, OAC, AF type, SBP at event time, CrCl, BMI, and hemoglobin	HR
**Chinese AF registry**	All-cause death	Age, BMI, AF type, HF, CAD, diabetes, and comprehensive baseline medications	HR
**ROCKET-AF**	Intracranial haemorrhage: any primary bleed into the cranial cavity that was clinically overt (caused symptoms or signs) and confirmed by CT or MRI brain imaging or autopsy.	race, randomisation to rivaroxaban, age, serum albumin, platelet count below 210×10^9/L, history of congestive heart failure, previous stroke or TIA, and diastolic blood pressure	HR

[a] contributed crude incidence rate ratios derived directly from reported stratum-specific event counts and follow-up time; no covariate adjustment was applied

[b] Covariate list verified against patient-level data available in the published supplementary material.

Supplementary Table S3. Contribution of each cohort to each analysis

	Systolic BP						Diastolic BP						Descriptive sections					
**Cohort**	**Mort**	**ICH**	**IS**	**S/SE**	**MB**	**CVD**	**Mort**	**IS**	**MB**	**S/SE**	**CVD**	**ICH**	**TTR**	**NCB**	**Dem**	**Age**	**LVEF**	**Renal**
Badheka 2014 (AFFIRM)	✓	—	—	—	—	—	✓	—	—	—	—	—	—	—	—	—	—	—
Bager 2021 (SPCCD)	✓	✓	✓	✓	—	—	—	—	—	—	—	—	—	—	—	—	—	—
Böhm 2020 (RE-LY)	✓	—	—	✓	✓	—	✓	—	✓	—	—	—	—	—	—	—	—	—
Ishida 2025 (F-CREATE)	—	✓	✓	—	—	—	—	—	—	—	—	—	—	—	—	—	—	—
Jeong 2026 (NHIS)	✓	—	✓	—	—	—	✓	✓	—	—	—	—	—	—	—	✓	—	—
Kim 2018 (NHIS)	—	✓	—	—	—	—	—	—	—	—	—	—	—	—	—	—	—	—
Kim 2020 (NHIS-NSC)	—	—	—	—	—	—	—	—	—	—	—	—	—	—	✓	—	—	—
Kim 2022 (Asan)	—	—	—	✓	—	—	—	—	—	✓	—	—	✓	—	—	—	—	—
Kodani 2022a (J-RISK AF)	✓	—	✓	—	✓	✓	✓	✓	✓	—	✓	—	—	—	—	—	—	—
Kodani 2022b (J-RHYTHM)	—	—	—	—	—	—	—	—	—	—	—	—	✓	—	—	—	—	—
Krittayaphong 2021 (COOL-AF)	✓	✓	✓	—	—	—	—	—	—	—	—	—	—	—	—	—	—	—
Krittayaphong 2024 (COOL-AF)	—	—	—	—	—	—	—	—	—	—	—	—	✓	—	—	—	—	—
Lip 2007 (SPORTIF III/V)	✓	—	—	✓	✓	—	✓	—	—	—	—	—	—	—	—	—	—	—
Minhas 2020 (CPRD)	✓	—	—	—	—	✓	—	—	—	—	—	—	—	—	—	—	—	—
Shao 2022 (MIMIC-III)	✓	—	—	—	—	—	—	—	—	—	—	—	—	—	—	—	—	—
Xu 2023 (Chinese AF)	✓	—	—	—	—	—	✓	—	—	—	—	—	—	—	—	—	—	—
Tremblay-Gravel 2014 (AFFIRM + AF-CHF)	—	—	—	—	—	—	—	—	—	—	—	—	—	—	—	—	✓	—
Shantsila 2024 (AF-GEN-UK)	✓	—	—	—	—	—	—	—	—	—	—	—	—	—	—	—	—	✓

	**Systolic BP**						**Diastolic BP**						**Descriptive sections**					
**Cohort**	**Mort**	**ICH**	**IS**	**S/SE**	**MB**	**CVD**	**Mort**	**IS**	**MB**	**S/SE**	**CVD**	**ICH**	**TTR**	**NCB**	**Dem**	**Age**	**LVEF**	**Renal**
Vemulapalli 2016 (ROCKET AF)	(c)	(c)	(c)	(c)	(c)	(c)	—	—	—	—	—	—	—	—	—	—	—	—
Vemulapalli 2019 (ORBIT-AF)	—	—	—	(c)	(c)	—	—	—	—	—	—	—	—	—	—	—	—	—
Hankey 2014 (ROCKET AF)	—	—	—	—	—	—	—	—	—	—	—	(c)	—	—	—	—	—	—
Park 2019 (ENGAGE AF-TIMI 48)	—	—	—	—	—	—	—	—	—	—	—	—	—	✓	—	—	—	—
Kario 2023 (ANAFIE)	—	—	—	—	—	—	—	—	—	—	—	—	—	✓	—	—	—	—

✓ = categorical strata contributing to the dose-response (spline) model, or the cohort informing that descriptive section. (c) = continuous estimate contributing to the per-increment pairwise analysis only. Mort = all-cause mortality; ICH = intracranial haemorrhage; IS = ischaemic stroke; S/SE = stroke or systemic embolism; MB = major bleeding; CVD = cardiovascular death; TTR = systolic blood pressure time in target range; NCB = net clinical benefit; Dem = dementia; Age = age-stratified analysis; LVEF = left ventricular ejection fraction; Renal = renal function.

Supplementary Table S4. Analyses by exposure and outcome, with contributing cohorts

Exposure	Analysis	k	Contributing cohorts
SBP	All-cause mortality	11	Badheka; Bager; Böhm; Jeong; Kodani 2022a; Krittayaphong 2021; Lip; Minhas; Shao; Xu ; Shinstila
SBP	Ischaemic stroke	5	Bager; Ishida; Jeong; Kodani 2022a; Krittayaphong 2021
SBP	Intracranial haemorrhage	4	Bager; Ishida; Kim 2018; Krittayaphong 2021
SBP	Stroke / systemic embolism	4	Bager; Böhm; Kim 2022; Lip
SBP	Major bleeding	3	Böhm; Kodani 2022a; Lip
SBP	Cardiovascular death	2	Kodani 2022a; Minhas — not modelled
DBP	All-cause mortality	6	Badheka; Böhm; Jeong; Kodani 2022a; Lip; Xu
DBP	Ischaemic stroke	2	Jeong; Kodani 2022a — not modelled
DBP	Major bleeding	2	Böhm; Kodani 2022a — not modelled
DBP	Stroke / systemic embolism	1	Kim 2022 — not modelled
DBP	Cardiovascular death	1	Kodani 2022a — not modelled
DBP	Intracranial haemorrhage	1	Hankey (continuous only) — not modelled

Dose-response models were fitted only where at least three cohorts contributed categorical data. Outcomes marked “not modelled” are reported descriptively.

Figure S1. Assessment of risk of bias using the ROBINS-E risk of tool for non-randomised observational cohorts

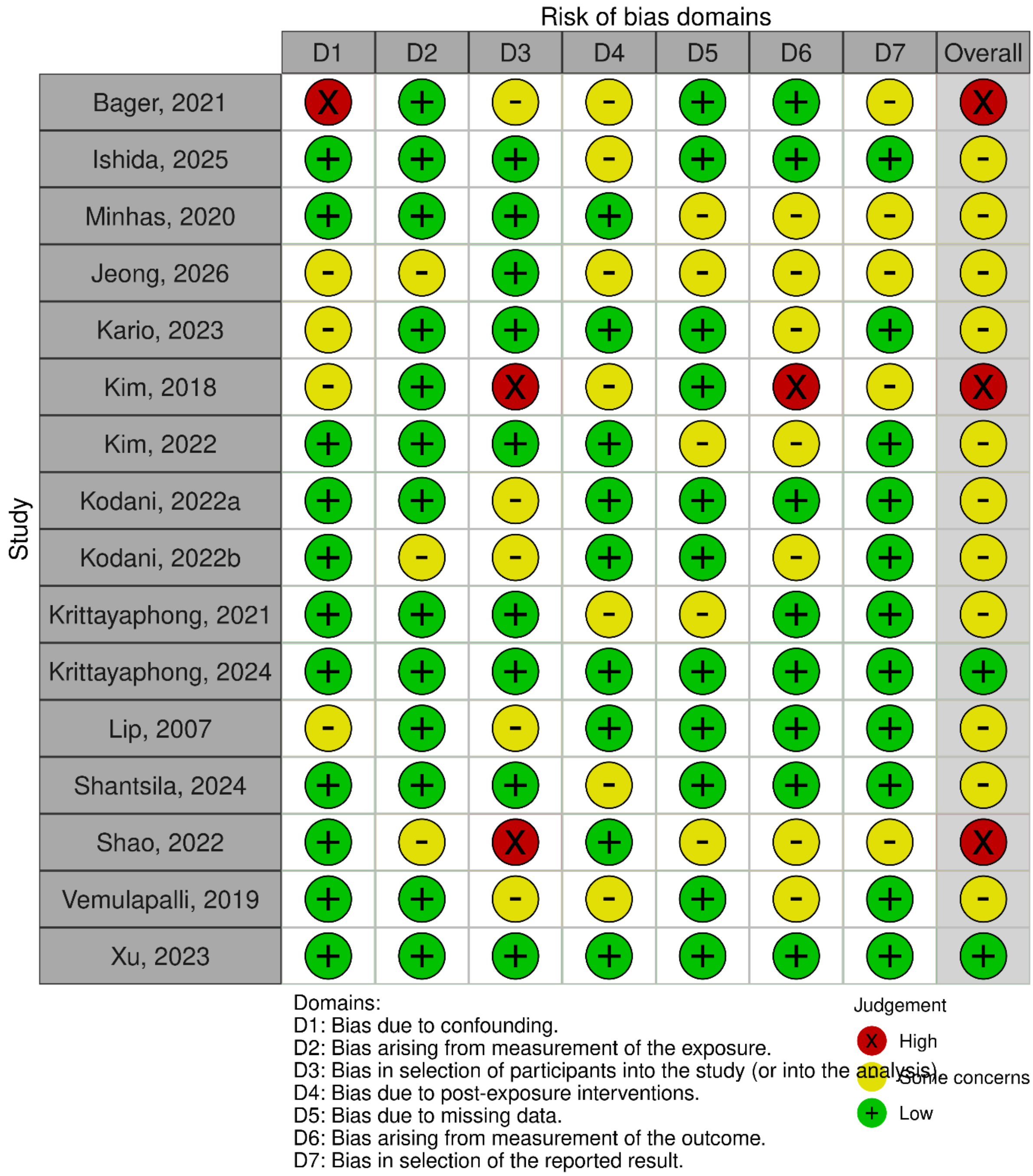

Figure S2. Assessment of risk of bias using the ROB-2 risk of tool for randomised observational cohorts

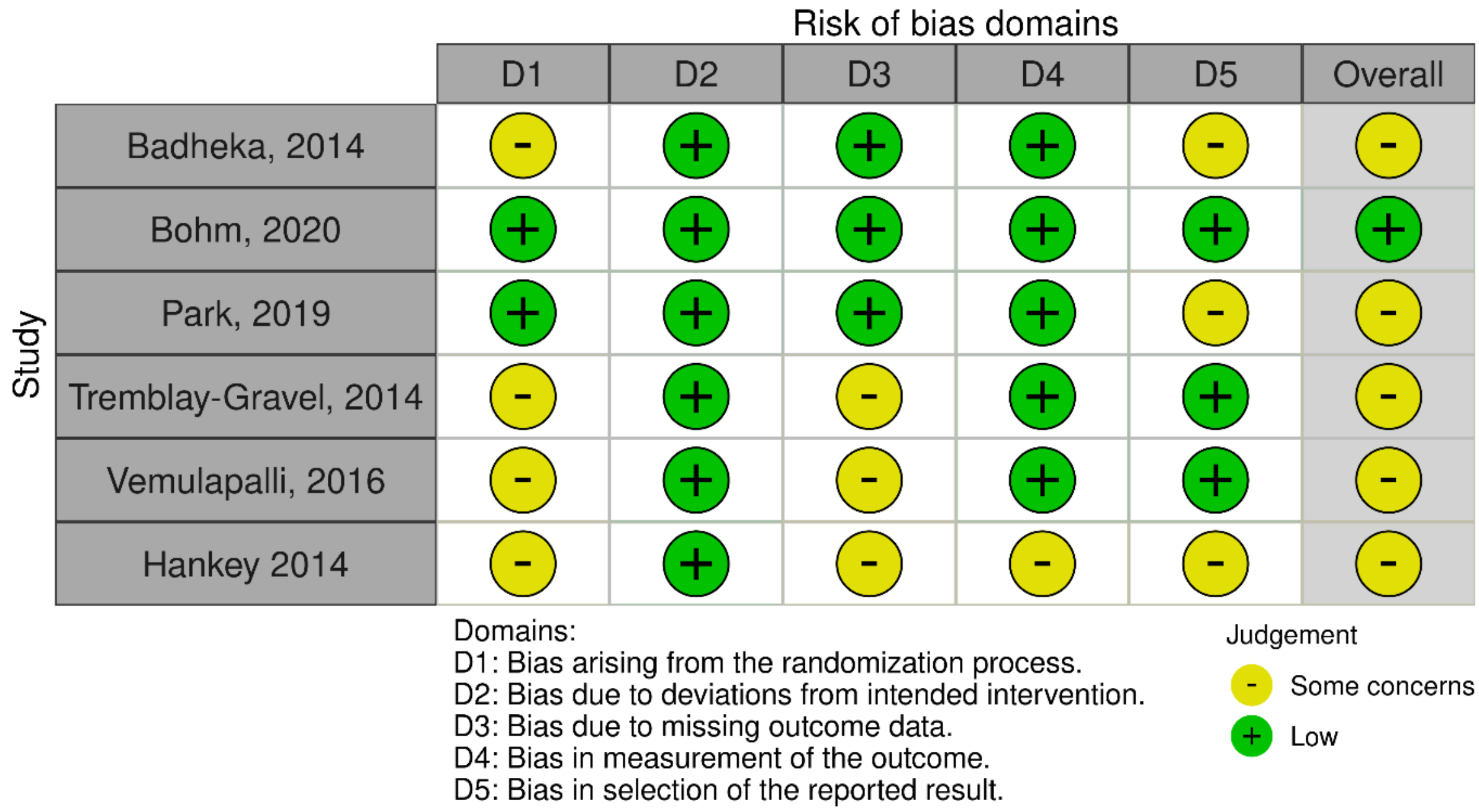

Supplementary Table S5. Model characteristics and fitted curve shape, systolic blood pressure.

Outcome	Cohorts	Participants	Events	Covariance	P nonlin.	Shape	Nadir (95% CI)
Intracranial haemorrhage	4	169,540	4,317	GL	<0.001	Monotonic increase	—
Ischaemic stroke	5	365,161	39,966	GL	<0.001	Monotonic increase	—
Stroke or systemic embolism	4	42,817	1,166	Independent	0.002	Monotonic increase	—
Major bleeding	3	43,080	535	Independent	0.002	Non-linear; no level significantly different from referent	133 mm Hg; 0.95 (0.82–1.10)
All-cause mortality	11	407,885	108,588	Independent	<0.001	U-shaped	134 mm Hg; 0.92 (0.86–0.98)

GL = Greenland–Longnecker approximation of within-study covariances. Independence was assumed where contributing cohorts did not report events per blood-pressure stratum A nadir is reported only where the fitted curve has an interior minimum.

Supplementary Table S6. Pooled relative risk per 10 mm Hg increase in systolic blood pressure, with heterogeneity.

Outcome	Studies	RR per 10 mm Hg (95% CI)	I^2 (95% CI)	τ^2	P (Q)
Intracranial haemorrhage	5	1.20 (1.01–1.43)	82% (43–98)	0.015	<0.001
Stroke or systemic embolism	6	1.10 (1.05–1.16)	53% (0–92)	0.001	0.063
Ischaemic stroke	6	1.07 (1.00–1.13)	55% (0–97)	0.001	0.026
Major bleeding	5	1.00 (0.94–1.07)	73% (18–98)	0.002	0.012
Cardiovascular death	3	0.93 (0.87–1.00)	1% (0–98)	<0.001	0.431
All-cause mortality	11	Not pooled (U-shaped)	—	—	—

Random-effects estimates with confidence intervals by the Hartung-Knapp-Sidik-Jonkman method; τ^2 and I^2 by restricted maximum likelihood, with 95% CIs.

Supplementary Figure S3. Leave-one-out sensitivity analysis for All-cause mortality associated with systolic blood pressure

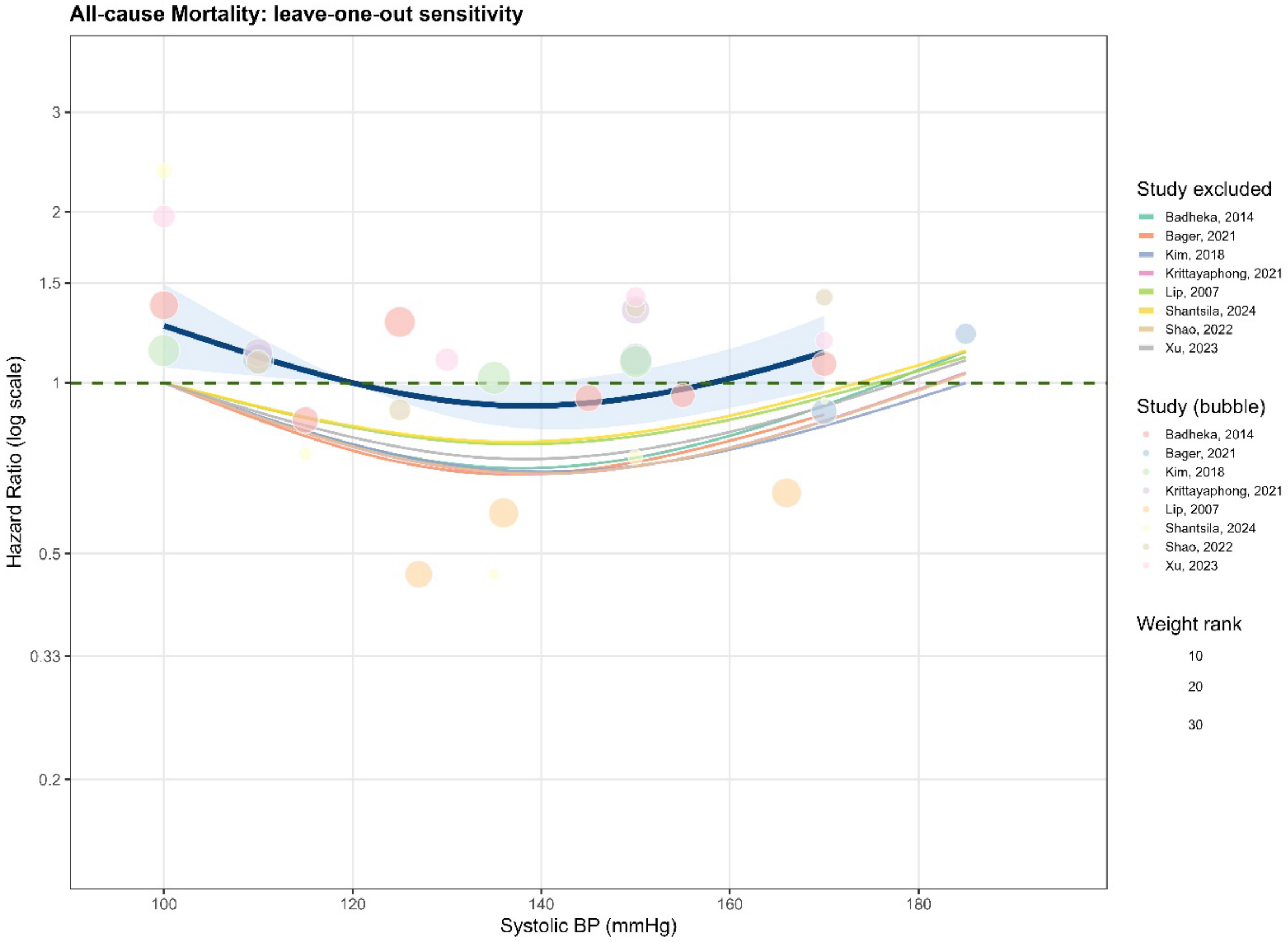

Supplementary Figure S4. Funnel plots

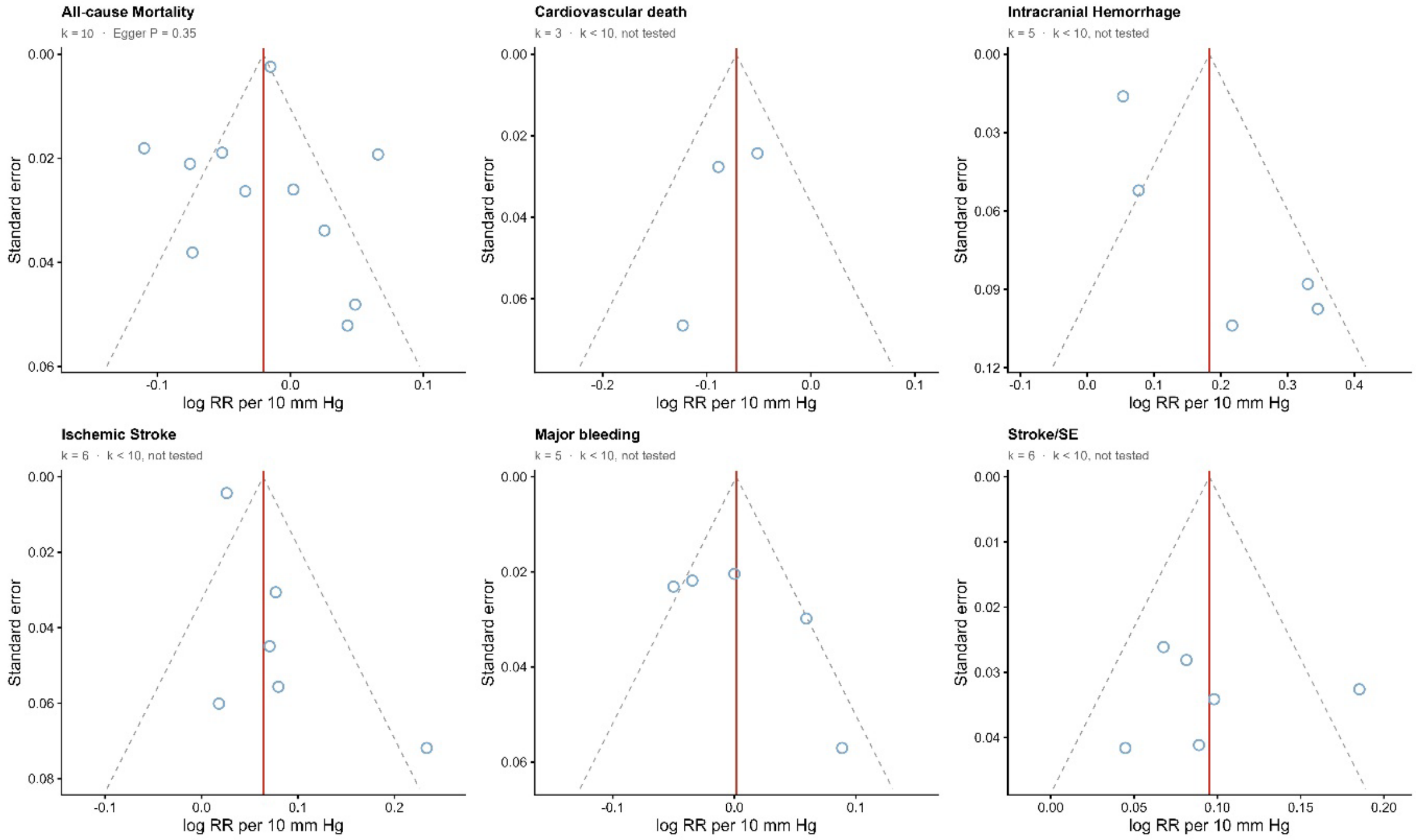

Supplementary Figure S5. Sensitivity Analysis Restricting to Studies Reporting Hazard Ratios

Dose-response associations between systolic blood pressure and clinical outcomes in atrial fibrillation, restricted to studies reporting multivariable-adjusted hazard ratios (HR) from Cox proportional hazards models (Badheka 2014, Shao 2022, and Lip 2007 excluded). The solid blue line represents the pooled random-effects natural cubic spline; the shaded band is the 95% confidence interval. All HRs are expressed relative to SBP 120 mmHg. Open circles represent individual study strata, sized proportionally to precision (1/SE).

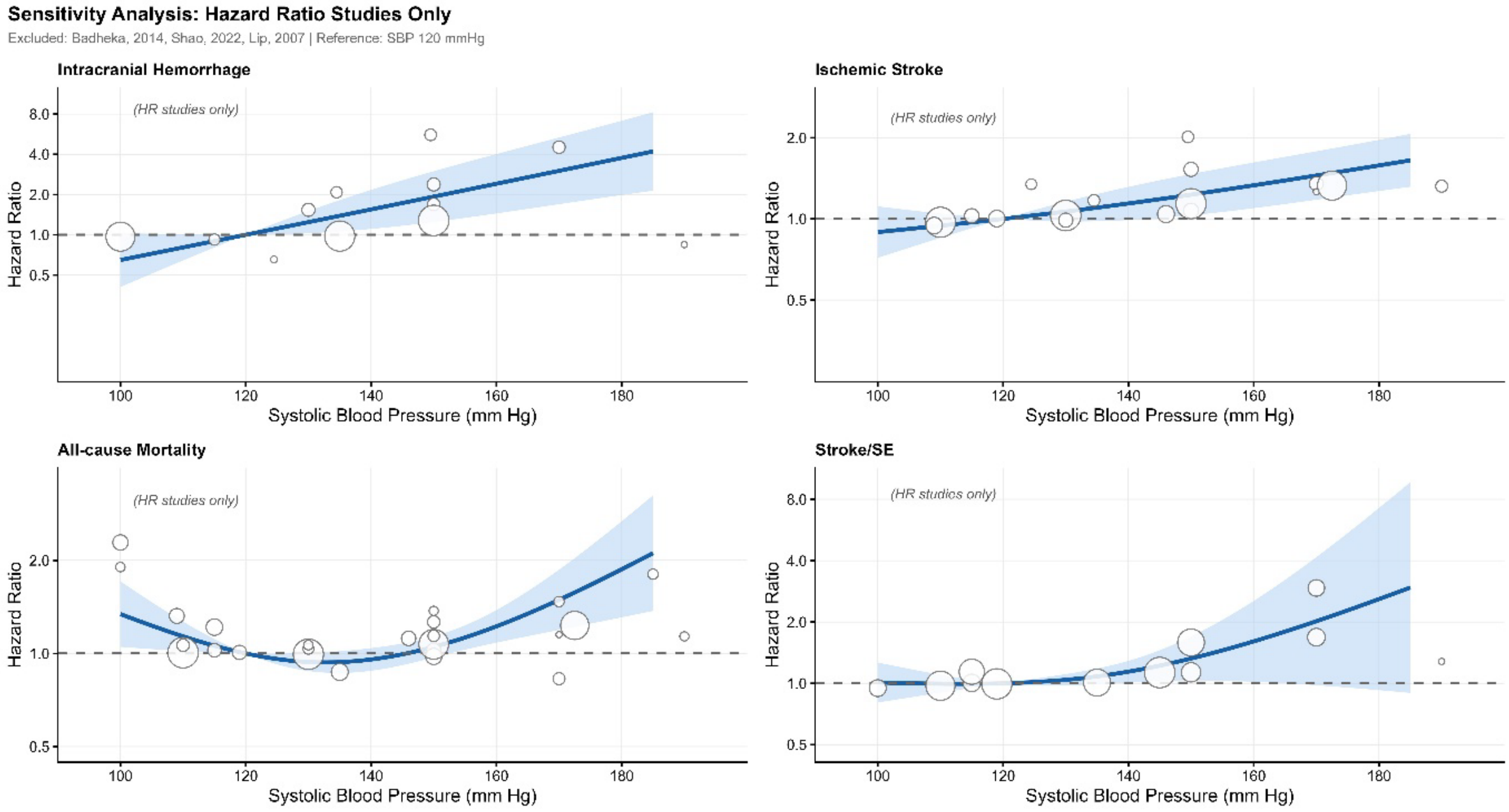

Supplementary Table S7. Pooled Hazard Ratios (95% CI) at Key SBP Thresholds: Sensitivity Analysis Restricted to Hazard Ratio Studies

Outcome	SBP 100	SBP 110	SBP 120	SBP 130	SBP 140	SBP 150	SBP 160	SBP 170
**Intracranial Hemorrhage**	0.65 (0.41–1.03)	0.80 (0.64–1.01)	**1.00 (ref)**	1.24 (1.03–1.50)	1.55 (1.11–2.16)	1.93 (1.25–2.99)	2.41 (1.45–4.01)	3.01 (1.69–5.34)
**Ischemic Stroke**	0.89 (0.72–1.11)	0.94 (0.85–1.05)	**1.00 (ref)**	1.06 (0.98–1.16)	1.14 (0.99–1.31)	1.23 (1.03–1.47)	1.33 (1.10–1.62)	1.45 (1.18–1.78)
**All-cause Mortality**	1.34 (1.05–1.71)	1.14 (1.02–1.27)	**1.00 (ref)**	0.94 (0.88–1.00)	0.96 (0.88–1.04)	1.05 (0.97–1.14)	1.22 (1.08–1.38)	1.49 (1.19–1.86)
**Stroke/Systemic Embolism**	1.01 (0.81–1.27)	1.00 (0.90–1.10)	**1.00 (ref)**	1.04 (0.98–1.11)	1.14 (1.01–1.29)	1.32 (1.03–1.70)	1.61 (1.02–2.54)	2.02 (0.98–4.16)

Estimates from a two-stage random-effects natural cubic spline (centred at SBP 120 mmHg). Restricted to studies reporting multivariable-adjusted hazard ratios from Cox proportional hazards models. Excluded: Badheka 2014, Shao 2022, Lip 2007

Supplementary Table S8. Pooled relative risk (95% CI) for all-cause mortality at key diastolic blood pressure thresholds, referent 80 mm Hg

**DBP, mm Hg**	**55**	**60**	**65**	**70**	**75**	**80**	**85**	**90**	**100**
All-cause mortality	1.49 (1.15–1.92)	1.36 (1.11–1.65)	1.24 (1.08–1.43)	1.13 (1.04–1.23)	1.04 (1.01–1.08)	1.00 (ref)	1.00 (0.96–1.04)	1.02 (0.93–1.11)	1.09 (0.90–1.32)

Supplementary Table S9. Cohorts contributing diastolic blood pressure data, by outcome

Outcome	Cohorts	Participants	Per 5 mm Hg (95% CI)
All-cause mortality	6	388,573	Not pooled (J-shaped)
Intracranial haemorrhage	1	14,264	1.08 (1.00–1.17)
Stroke or systemic embolism	1	9,505	1.05 (0.99–1.10)

Supplementary Table S10. Study characteristics of cohorts contributing to systolic blood pressure time-in-target-range analyses

Characteristic	Kim et al., 2022	COOL-AF, 2024	Kodani et al., 2022
**Country**	South Korea	Thailand	Japan
**Study design**	Retrospective cohort (nationwide registry)	Prospective registry (COOL-AF)	Prospective registry
**Follow-up**	Median 2.7 yr (IQR 1.1–4.9)	Mean 32.1 ± 8.3 months	2 years
**TTR target (SBP)**	< 130 mmHg	120–140 mmHg	110–130 and 120–140 mmHg
**TTR target (DBP)**	< 80 mmHg	Not reported	Not reported
**TTR grouping**	Quartiles (Q1–Q4)	Tertiles (T1–T3)	Fixed cut-offs (< 25%, 25–49.9%, 50–74.9%, ≥ 75%)
**Reference group**	Highest TTR (Q4, ≥ 94%)	Lowest TTR (T1, < 32.84%)	Highest TTR (≥ 75%)
**TTR method**	Rosendaal linear interpolation	Rosendaal linear interpolation	Not specified
**Primary outcome(s)**	Ischaemic stroke / systemic embolism (SSE)	All-cause death, SSE, major bleeding, heart failure	Thromboembolism, major bleeding, all-cause death, CV death
**Multivariable adjustment**	Yes (age, sex, CHA_2DS_2-VASc, anticoagulation)	Yes (all baseline variables)	Model 1 (unadjusted) and Model 2 (fully adjusted)

IQR = interquartile range; SBP = systolic blood pressure; SSE = ischaemic stroke or systemic embolism; TTR = time in target range.

Supplementary Table S11. SSE / thromboembolic outcomes by SBP-TTR group

Study / target	Comparison	Adj. HR (95% CI)	P-value
**Kim 2022 — SBP < 130 mmHg**	Q3 vs Q4 (ref)	1.33 (1.08–1.63)	0.007
	Q2 vs Q4 (ref)	1.43 (1.16–1.75)	< 0.001
	Q1 vs Q4 (ref)	1.55 (1.27–1.89)	< 0.001
**Kim 2022 — DBP < 80 mmHg**	Q2 vs Q3+Q4 (ref)	1.22 (1.01–1.48)	0.04
	Q1 vs Q3+Q4 (ref)	1.42 (1.16–1.74)	< 0.001
**COOL-AF 2024 — SBP 120–140 mmHg**	T2 vs T1 (ref)	0.64 (0.42–0.98)	0.04
	T3 vs T1 (ref)	0.86 (0.57–1.30)	0.47
**Kodani 2022 — SBP 110–130 mmHg**	< 25% vs ≥ 75% (ref), Model 1	0.80 (0.45–1.44)	0.246
	< 25% vs ≥ 75% (ref), Model 2	0.66 (0.35–1.23)	0.187
**Kodani 2022 — SBP 120–140 mmHg**	< 25% vs ≥ 75% (ref), Model 1	1.53 (0.82–2.84)	0.179
	< 25% vs ≥ 75% (ref), Model 2	1.38 (0.69–2.76)	0.365

Adjusted HRs are from multivariable Cox proportional-hazards models

Supplementary Table S12. All-cause and cardiovascular mortality outcomes by SBP-TTR group

Study / target	Outcome	Group	Events	Rate /100 py	Adj. HR (95% CI)	P
**COOL-AF 2024 — SBP 120–140 mmHg**	All-cause death	T1 (< 32.84%) — ref	155	5.37	1.00 (reference)	—
		T2 (32.85–60.84%)	104	3.38	— (not reported)	—
		T3 (≥ 60.85%)	91	3.03	0.62 (0.48–0.80)	<0.001
**Kodani 2022 — SBP 110–130 mmHg**	All-cause death (Model 2)	< 25% vs ≥ 75% (ref)	—	—	1.36 (0.77–2.40)	0.291
		25–49.9% vs ≥ 75% (ref)	—	—	1.66 (0.95–2.90)	0.074
		50–74.9% vs ≥ 75% (ref)	—	—	1.07 (0.59–1.94)	0.835
**Kodani 2022 — SBP 120–140 mmHg**	All-cause death (Model 2)	< 25% vs ≥ 75% (ref)	—	—	1.31 (0.65–2.63)	0.447
		25–49.9% vs ≥ 75% (ref)	—	—	2.05 (1.05–4.02)	0.035
		50–74.9% vs ≥ 75% (ref)	—	—	1.87 (0.94–3.73)	0.074
**Kodani 2022 — SBP 110–130 mmHg**	CV death (Model 2)	< 25% vs ≥ 75% (ref)	—	—	2.37 (0.85–6.66)	0.101
		25–49.9% vs ≥ 75% (ref)	—	—	2.02 (0.70–5.86)	0.194

Kim et al. 2022 did not report mortality outcomes. COOL-AF incidence rates are directly published; Kodani 2022 exact event counts per TTR group were not separately reported for mortality. CV = cardiovascular; py = person-years; T = tertile; TTR = time in target range.

Supplementary Table S13. Intracranial haemorrhage and Ischemic stroke, stratified by blood pressure control , and calculated net clinical benefit

Study	SBP stratum (mmHg)	Stroke, NOAC	Stroke, warfarin	ICH, NOAC	ICH, warfarin	NCB (NOAC vs warfarin)	Favored strategy
ANAFIE	115–124	1.00	1.31	0.55	0.59	+0.37	NOAC
ANAFIE	125–134	1.38	1.44	0.52	0.32	−0.24	Warfarin
ANAFIE	135–144	1.16	1.18	0.49	0.58	+0.16	NOAC
ANAFIE	145–165	1.88	3.39	1.31	3.43	+4.69	NOAC
ENGAGE AF-TIMI 48	100–119	0.59	0.79	0.35	0.64	+0.64	NOAC
ENGAGE AF-TIMI 48	120–129	0.74	1.15	0.21	0.62	+1.03	NOAC
ENGAGE AF-TIMI 48	130–139	1.26	1.24	0.32	0.63	+0.45	NOAC
ENGAGE AF-TIMI 48	140–149	1.42	2.08	0.67	1.37	+1.71	NOAC
ENGAGE AF-TIMI 48	150–170	1.59	2.42	0.67	1.37	+1.88	NOAC

DBP, mm Hg	Ischaemic stroke, NOAC	Ischaemic stroke, warfarin	ICH, NOAC	ICH, warfarin	Net clinical benefit	Favours
<75	0.61	0.72	0.39	0.88	+0.84	NOAC
75–<85	0.75	0.64	0.24	0.59	+0.41	NOAC
85–<90	0.47	1.04	0.62	0.75	+0.77	NOAC
≥90	1.76	2.66	0.50	1.68	+2.67	NOAC

Supplementary Results

Four prespecified secondary analyses were each informed by a single cohort and could not be pooled. They are reported in full below and summarised in the main text under 'Analyses informed by single cohorts'. Because each rest on one cohort, these findings are hypothesis-generating rather than confirmatory.

Supplementary Table S14. Blood pressure and incident dementia in a single cohort

Analysis	Comparison	Estimate (95% CI)
Continuous, above nadir	Per 10 mm Hg increase above SBP 120 mm Hg	4.4% higher risk (2.7 to 6.0)
Continuous, below nadir	Per 10 mm Hg decrease below SBP 120 mm Hg	4.6% higher risk (0.1 to 8.2)
Time-updated category	SBP <120 / DBP <80 vs 120–129 / 80–84 mm Hg	HR 1.08 (1.02–1.14)
Time-updated category	SBP 130–139 / DBP 85–89 vs 120–129 / 80–84 mm Hg	HR 1.09 (1.02–1.15)
Time-updated category	SBP ≥140 / DBP ≥90 vs 120–129 / 80–84 mm Hg	HR 1.07 (1.01–1.13)

Kim et al. (2020), Korean National Health Insurance Service; n = 171,228 patients with atrial fibrillation aged 50–69 years; mean follow-up 6.6 years; 9,909 incident dementia events. The association between systolic pressure and overall dementia risk was U-shaped with a nadir at SBP 120 mm Hg.

Supplementary Table S15. Age-stratified associations of blood pressure with ischaemic stroke and all-cause mortality in a single cohort

Outcome	Contrast	Age 40–49 years	Age ≥ 70 years	P for interaction
Ischaemic stroke	SBP ≥160 vs <100 mm Hg	3.35 (1.94–5.78)	1.15 (1.02–1.29)	<0.001
Ischaemic stroke	SBP 140–160 vs <100 mm Hg	Not reported	1.05 (0.94–1.17)	
Ischaemic stroke	DBP ≥100 vs <80 mm Hg	2.05 (1.62–2.61)	1.31 (1.24–1.39)	
All-cause mortality	SBP 120–140 vs <100 mm Hg	Not reported	0.85 (0.81–0.90)	<0.001
All-cause mortality	SBP ≥160 vs <100 mm Hg	Not reported	0.97 (0.91–1.03)	

Jeong et al. (2026), Korean National Health Insurance Service; n = 338,747. Contributing age bands: 40–49 years, n = 25,284 (1,619 deaths; 811 ischaemic strokes); 50–59 years, n = 60,500 (6,389; 3,681); 60–69 years, n = 93,967 (18,867; 9,508); ≥ 70 years, n = 158,996 (77,837; 25,329).

.

Outcome	DBP, mm Hg	40–49 y	50–59 y	60–69 y	≥ 70 y
Ischaemic stroke	<80 (referent)	1.00 (ref)	1.00 (ref)	1.00 (ref)	1.00 (ref)
Ischaemic stroke	90–<100	1.50 (1.20–1.87)	Not extracted	Not extracted	1.19 (1.15–1.24)
Ischaemic stroke	≥100	2.05 (1.62–2.61)	Not extracted	Not extracted	1.31 (1.24–1.39)
All-cause mortality	<80 (referent)	1.00 (ref)	1.00 (ref)	1.00 (ref)	1.00 (ref)
All-cause mortality	≥100	Not extracted	1.25 (1.13–1.40)	Not extracted	1.16 (1.12–1.20)

Supplementary Table S16. Effect modification of the systolic blood pressure–mortality association by left ventricular ejection fraction in a single post-hoc analysis

Subgroup	Outcome	Contrast	Estimate (95% CI)
Preserved LVEF (>40%)	All-cause mortality	Systolic pressure, overall association	No association (P = 0.563)
Preserved LVEF (>40%)	Cardiovascular mortality	Systolic pressure, overall association	No association (P = 0.087)
Reduced LVEF (≤40%)	All-cause mortality	SBP <120 vs 120–140 mm Hg	HR 1.75 (1.41–2.17)
Reduced LVEF (≤40%)	All-cause mortality	SBP >140 vs 120–140 mm Hg	HR 1.40 (1.04–1.90)
Reduced LVEF (≤40%)	Cardiovascular mortality	SBP <120 and >140 vs 120–140 mm Hg	Concordant U-shaped pattern

Tremblay-Gravel et al. (2014), post-hoc analysis pooling the AFFIRM and AF-CHF trials; n = 5,436. The association between systolic pressure and mortality was U-shaped in patients with reduced ejection fraction and absent in those with preserved ejection fraction.

Supplementary Table S17. Renal function, mortality and blood pressure in a single registry

Analysis	Outcome	Estimate (95% CI)
eGFR, univariate	All-cause death	HR 0.99 (0.98–0.996)
eGFR, adjusted*	All-cause death	HR 1.00 (0.98–1.01)
eGFR × SBP category interaction	All-cause death; thromboembolism	P > 0.05
eGFR × DBP category interaction	All-cause death; thromboembolism	P > 0.05

*Shantsila et al. (2024), AF-GEN-UK registry; n = 1402. Renal function was broadly preserved across systolic blood pressure categories and severe impairment (eGFR <30 mL/min) was uncommon at every level. *Adjusted for age, oral anticoagulation and CHA2DS2-VASc score.*

Supplementary Table S18. Exploratory subgroup analyses and meta-regression for the systolic blood pressure and mortality association

Limb / moderator	Subgroup	RR per 10 mmHg (95% CI)	I^2 (%)	Q	R^2 (%)	P
High limb (>120 mmHg)	k = 11	1.03 (0.99–1.07)	87			
Study design	Registry (k = 8)	1.05 (1.01–1.09)	81	1.08	–	0.299
	Trial (k = 3)	0.98 (0.87–1.11)	95		–	
Anticoagulant era	NOAC (k = 5)	1.06 (1.00–1.12)	89	1.46	–	0.228
	Warfarin (k = 6)	1.00 (0.93–1.07)	87		–	
BP measurement setting	Acute (k = 2)	1.07 (1.01–1.14)	0	0.88	–	0.644
	Outpatient (k = 6)	1.02 (0.93–1.11)	91		–	
	Primary care (k = 2)	1.05 (0.97–1.14)	84		–	
	Screening (k = 1)	0.99 (single study)	–		–	
Follow-up (per year)	k = 11	0.990 (0.963–1.018)	85	0.50	0	0.480
OAC use (per 10%)	k = 9	0.991 (0.972–1.010)	86	0.85	0	0.355
Low limb (<120 mmHg)	k = 9	0.82 (0.63–1.08)	97			
Study design	Registry (k = 7)	0.87 (0.67–1.13)	91	0.65	–	0.420
	Trial (k = 2)	0.71 (0.48–1.06)	95		–	
Anticoagulant era	NOAC (k = 4)	0.81 (0.52–1.28)	98	0.00	–	0.959
	Warfarin (k = 5)	0.82 (0.74–0.92)	4		–	
BP measurement setting	Acute (k = 2)	0.78 (0.61–0.99)	35	0.06	–	0.806
	Outpatient (k = 5)	0.75 (0.59–0.94)	88		–	
	Primary care (k = 1)	0.83 (single study)	–		–	
	Screening (k = 1)	1.19 (single study)	–		–	
Follow-up (per year)	k = 9	1.111 (1.034–1.194)	70	8.23	15	0.054
OAC use (per 10%)	k = 7	0.990 (0.931–1.054)	79	0.09	0	0.760
